# 2024 European Thyroid Association Guidelines on diagnosis and management of genetic disorders of thyroid hormone transport, metabolism and action

**DOI:** 10.1530/ETJ-24-0125

**Published:** 2024-08-03

**Authors:** Luca Persani, Patrice Rodien, Carla Moran, W Edward Visser, Stefan Groeneweg, Robin Peeters, Samuel Refetoff, Mark Gurnell, Paolo Beck-Peccoz, Krishna Chatterjee

**Affiliations:** 1 Department of Endocrine and Metabolic Diseases, IRCCS Istituto Auxologico Italiano, Milano, Italy; 2 Department of Medical Biotechnology and Translational Medicine, University of Milan, Milano, Italy; 3 Service d’Endocrinologie-Diabétologie-Nutrition, Centre de référence des maladies rares de la Thyroïde et des récepteurs hormonaux, CHU d’Angers, Angers, France.; 4 Institute of Metabolic Science, University of Cambridge, Cambridge, UK; 5 Endocrine Section, Beacon Hospital, Dublin, Ireland.; 6 School of Medicine, University College Dublin, Ireland; 7 Endocrinology Department, St Vincent’s University Hospital, Dublin, Ireland; 8 Department of Internal Medicine and Rotterdam Thyroid Center, Erasmus University Medical Center, Rotterdam, The Netherlands; 9 Departments of Medicine and Paediatrics and Committee on Genetics, The University of Chicago, Chicago, Illinois, USA

**Keywords:** clinical practice guideline, deiodinase, diagnosis and management, impaired sensitivity to thyroid hormones, resistance to thyroid hormone, selenoprotein, thyroid hormone receptor, thyroid hormone transporter

## Abstract

Impaired sensitivity to thyroid hormones encompasses disorders with defective transport of hormones into cells, reduced hormone metabolism, and resistance to hormone action. Mediated by heritable single-gene defects, these rare conditions exhibit different patterns of discordant thyroid function associated with multisystem phenotypes. In this context, challenges include ruling out other causes of biochemical discordance, making a diagnosis using clinical features together with the identification of pathogenic variants in causal genes, and managing these rare disorders with a limited evidence base. For each condition, the present guidelines aim to inform clinical practice by summarizing key clinical features and useful investigations, criteria for molecular genetic diagnosis, and pathways for management and therapy. Specific, key recommendations were developed by combining the best research evidence available with the knowledge and clinical experience of panel members, to achieve a consensus.

## Introduction

Membrane transporters are rate-limiting for cellular entry of thyroid hormones (TH; T4 and T3) into some tissues, with selenocysteine-containing deiodinase enzymes (D1, D2) converting T4 to the biologically active hormone T3. THs exert their physiological effects principally by regulating the expression of target genes via hormone-inducible nuclear receptors (TRα and TRβ).

A consensus statement after the 12th International Workshop on resistance to thyroid hormone widened the definition of disorders with impaired sensitivity to thyroid hormones to include conditions with defective cellular uptake of TH via membrane transporters, reduced intracellular hormone metabolism generating T3 from T4, as well as resistance to thyroid hormone action via nuclear receptors (1, 2, 3).

Accordingly, these guidelines focus on the diagnosis and management of genetic disorders of thyroid hormone transport, metabolism, and action comprising resistance to thyroid hormone β (RTHβ), resistance to thyroid hormone α (RTHα), monocarboxylate transporter 8 (MCT8) defects, selenoprotein deficiency, and iodothyronine deiodinase 1 defects.

## Methodology and grading of evidence

Following consultation with its Guidelines Board, the Executive Committee of the ETA commissioned the development of this guideline by a team of experts led by one chairperson (KC). For each disorder, designated task force members with knowledge and expertise of this condition (one or more primary reviewers), were assigned. Following a review of the literature based on a systematic search of the MEDLINE database via the PubMed search engine, primary reviewers formulated clinical features and key investigations, guidance on diagnosis and management, together with statements of key recommendations. This information was assessed by secondary reviewers, with dialogue between primary and secondary reviewers, amending the guidance. Finally, the guidance was further refined (and amended if necessary) by all members of the task force to achieve a common consensus.

We have used the GRADE system to assess and sort out the quality of the evidence (4). The strength of each statement has been classified as strong (S, a recommendation or clinically important best practice applicable to most patients) or weak (W, a suggestion – to be considered by the clinician and applicable best practice in particular patients or contexts). The quality of the evidence concerning each aspect of the statement has been graded as: level 1, high quality (RCT evidence/meta-analysis (ØØØØ)); level 2, moderate quality (intervention short of RCT or large observational studies (ØØØO)); level 3, low quality (case-control studies, case series (ØØOO)); level 4, very-low quality (case reports, expert opinion (ØOOO)) using modified GRADE criteria (5). Boxes 1, 2, 3, and 4 summarise key recommendations for differential diagnosis of raised thyroid hormones and non-suppressed TSH as well as the diagnosis, management, and treatment of each disorder.

**Box 1. Summary of key recommendations – Differential diagnosis**

Differential diagnosis of raised thyroid hormones and non-suppressed TSH

- Potential confounding effects of medications and intercurrent illness should be carefully considered when assessing a patient with raised thyroid hormone concentrations and non-suppressed TSH (**Recommendation: S; Quality of evidence:** ØØOO).

- Laboratory assay interference in the measurement of thyroid hormones (T4, T3) and TSH should be excluded before screening for rare genetic and acquired disorders of thyroid hormone transport, metabolism, and action (**Recommendation: S; Quality of evidence:** ØØØO).
- Dynamic endocrine tests (TRH stimulation (if available), L-T3 suppression), pituitary imaging (MRI, PET), and trial of depot somatostatin receptor ligand may aid in the distinction of RTHβ and thyrotropinoma (**Recommendation: S; Quality of evidence:** ØØOO).

**Box 2. Summary of key recommendations – Resistance to thyroid hormone**

Recommendations in resistance to thyroid hormone β

*Diagnosis*

- We**recommend** considering a diagnosis of RTHβ in cases with a discordant pattern of thyroid function tests (TFTs), comprising genuinely elevated, circulating free (or total) T4, raised free (or total) T3, and nonsuppressed TSH (**Recommendation: S; Quality of evidence**: ØØ ØØ).
- Werecommendsuspecting RTHβ only when failure to normalize TSH in hypothyroidism is accompanied by high FT4 (**Recommendation: S; Quality of evidence:** ØØØO).
- If RTHβ is suspected, we **recommend** measuring TFTs in first-degree relatives. Finding abnormal TFTs in first-degree relatives strengthens the likelihood of RTHβ (**Recommendation: S; Quality of evidence:** ØØ ØØ).
- We **recommend** *THRB* sequencing to confirm the diagnosis of RTHβ in cases with genuinely raised thyroid hormones (T4 and T3) with nonsuppressed TSH (**Recommendation: S; Level of evidence:** ØØ ØØ).
- When a *THRB* variant of unknown significance (VUS) is identified by sequencing (e.g. next generation) in an index case or families, **we recommend** checking segregation with abnormal thyroid function tests in affected individuals to establish the pathogenicity and confirm RTHβ (**Recommendation: S; Quality of evidence:** ØØØO).
- In cases with a VUS in *THRB* where this is not possible (e.g. sporadic cases, unavailable family members, borderline abnormalities in thyroid function), **we suggest** *in vivo* assessment with a T3 suppression test as well as functional studies of the TRβ variant *in vitro* (less sensitive) (**Recommendation: W; Quality of evidence:** ØØOO).
- Following the diagnosis of RTHβ in an index case, **we recommend** testing (biochemical, then genetic) of first-degree relatives (**Recommendation: S; Quality of evidence:** ØØ ØØ).
- If a *THRB* mutation is absent with conventional sequencing (especially in cases with the transmission of abnormal thyroid function to progeny), **we recommend** next-generation sequencing of tissues other than blood to exclude RTHβ due to somatic mosaicism (**Recommendation: S; Quality of evidence:**ØOOO).

*Management*

- We **recommend** ultrasound evaluation of the thyroid/goiter (undertaken by a specialist with expertise in the classification (e.g. TIRADS) of thyroid nodules) at diagnosis and periodically thereafter (**Recommendation: S; Quality of evidence:** ØØ ØØ).
- We **recommend**the management of any thyroid nodule using standard practice (**Recommendation: S; Quality of evidence:** ØØØO).
- We **recommend** the assessment of concurrent anti-thyroid antibodies (Anti-TPO/TRAb) at diagnosis and during follow-up, whenever a change in thyroid status (circulating TSH or free TH) or symptoms (hypothyroid/thyrotoxic) is observed (**Recommendation: S; Quality of evidence:** ØØOO).
- In childhood, **we suggest**a careful evaluation of growth and development by a pediatric endocrinologist (**Recommendation: W; Quality of evidence:**ØOOO).
- We **suggest** treating ear, nose, and throat infections promptly to avoid or reduce complications (**Recommendation: W; Quality of evidence:** ØØOO).
- At diagnosis, **we suggest** undertaking audiometry to detect hearing deficit (**Recommendation: W; Quality of evidence:** ØØOO).
- When RTHβ is diagnosed in children, **we suggest** neuropsychological assessment and psychometric testing to diagnose ADHD and cognitive deficits that could cause learning difficulties (**Recommendation: W; Quality of evidence:** ØØOO).
- In children with RTHβ, **we suggest** considering educational support to manage learning disabilities and/or attention deficit hyperactivity disorder (**Recommendation: W; Quality of evidence:** ØØOO).
- In adult RTHβ patients, **we suggest** initial assessment and subsequent monitoring of bone density and markers of calcium homeostasis (serum calcium, PTH, 25OH-vitamin D) (**Recommendation: W; Quality of evidence:** ØØOO).
- We **recommend the** assessment of cardiovascular risk in all RTHβ patients over the age of 30 years (or younger in patients with cardiac symptoms or signs) at diagnosis, with an ongoing surveillance. This should include the measurement of blood pressure, electrocardiogram, and echocardiography, with cardiac telemetry and markers of cardiac function (e.g. NT-proBNP) in symptomatic cases (**Recommendation: S; Quality of evidence:** ØØØO).
- We **recommend** a metabolic assessment, including fasting lipids (total LDLc, HDLc, triglycerides), glucose, and HbA1c, in RTHβ patients at diagnosis, with periodic, ongoing monitoring (**Recommendation: S; Quality of evidence:** ØØØO).
- In RTHβ patients, **we suggest** monitoring the risk of metabolic dysfunction-associated steatotic liver disease (MASLD) (e.g. with fibroscan) (**Recommendation: W; Quality of evidence:** ØØOO).
- Due to increased risks (miscarriage, SGA, and LBW of neonates), **we suggest** multidisciplinary (endocrinologist, obstetrician) team management of all women with RTHβ embarking on pregnancy. (**Recommendation: W; Quality of evidence:** ØØOO).
- We **recommend** careful monitoring of fetal parameters (growth, heart rate) in all RTHβ women during pregnancy, with antithyroid drug treatment being considered in cases of fetal tachycardia or growth restriction (**Recommendation: S; Quality of evidence:** ØØOO).
- With the possible risk of SGA and LBW in normal offspring of mothers with RTHβ, **we suggest** considering antithyroid drug treatment during pregnancy when the maternal FT4 exceeds 150% of the upper limit of normal. Such intervention could be preceded by prenatal genetic diagnosis to identify unaffected fetuses, and this approach should ideally be undertaken in the context of a clinical trial (**Recommendation: W; Quality of evidence:**ØOOO).

*Treatment*

- We **recommend** avoiding treatment with antithyroid drugs or thyroid ablation (surgery or radioiodine) for RTHβ patients in the absence of significant comorbidities (see below) (**Recommendation: S; Quality of evidence:** ØØØO).
- We **recommend** limiting thyroid ablation to RTHβ cases with severe, uncontrolled, or life-threatening hyperthyroidism (e.g. heterozygous RTHβ and thyroid autonomy due to Graves’ disease/toxic nodule, or homozygous RTHβ with thyrotoxic cardiomyopathy) or large goiters with compression symptoms or thyroid malignancy. Post-ablation, levothyroxine therapy should aim to restore thyroid function tests to their original level (**Recommendation: S; Quality of evidence:** ØØOO).
- We **recommend** periodic, ongoing surveillance for comorbidities (thyroid autoimmunity, tachyarrhythmias, low bone density, dyslipidemia) (**Recommendation: S; Quality of evidence:** ØØØO).

- We **recommend** beta-blockade alone or, rarely, TRIAC therapy to control thyrotoxic symptoms (e.g. anxiety, tremor, palpitations) and signs (tachycardia) (**Recommendation: S; Quality of evidence:** ØØØO).
- We **recommend** that a decision to treat with TRIAC be made only made after discussion with centers with expertise in its use in this disorder (**Recommendation: S; Quality of evidence:** ØØØO).
- We **recommend** administering TRIAC twice or thrice daily. The dosage of TRIAC is titrated to control hyperthyroid symptoms and signs and lower circulating free T4 concentrations (TRIAC cross-reacts in immunoassays but not LC-MS/MS assays of T3) (**Recommendation: S; Quality of evidence:** ØØØO).
- We **recommend** considering cardioselective beta blockade to control tachycardia, atrial fibrillation, and supraventricular tachyarrhythmias in heterozygous RTHβ patients or cardiac hyperthyroidism in homozygous RTHβ (**Recommendation: S; Quality of evidence:** ØØOO).
- We **recommend** seeking expert cardiological advice on the management of atrial fibrillation in RTHβ, as chemical (e.g. amiodarone, flecainide) and electrical cardioversion or cardiac ablation may not be successful (**Recommendation: S; Quality of evidence:** ØØOO).
- We **suggest**that attention deficit-hyperactivity disorder, which interferes with daily activities of life, should be formally diagnosed by a neuropsychiatrist to decide on the most appropriate intervention. This can either be standard intervention or treatment with TRIAC (not yet licensed for RTHβ in all countries, but available on an individual basis) (**Recommendation: W; Quality of evidence:** ØØOO).
- In levothyroxine treatment of hypothyroidism (autoimmune or congenital) with coexisting RTHβ, elevated circulating TSH with normal free TH concentrations signify under replacement. In this context, we **recommend**that levothyroxine therapy be titrated to achieve FT4 concentrations comparable to other family members (with RTHβ alone) or maintain concurrent high-normal TSH and high free TH concentrations, monitoring cardiac function to avoid overtreatment (**Recommendation: S; Quality of evidence:** ØØOO).

**Recommendations in resistance to thyroid hormone α**

*Diagnosis*

- When considering a diagnosis of RTHα, we **recommend** full clinical assessment, including measurement of head circumference, standing height, sitting height, and subischial leg length, with a comparison of these results to age-appropriate population ranges to determine whether any of the recognized clinical phenotypes are present (**Recommendation: S, Quality of evidence:** ØØOO).
- If RTHα is suspected, we **suggest** undertaking additional investigations, which include (but are not limited to): full blood count, T4, T3, TSH, reverse T3, creatine kinase, skeletal and dental radiographs (**Recommendation: W, Quality of evidence:** ØØOO).
- We **recommend** that *THRA* sequencing be **performed** in any patient with:
  - three or more of the following major criteria: macrocephaly, short stature, constipation, typical biochemical profile (TSH within reference interval/mildly raised and low/low-normal T4 and raised/high-normal T3, with low reverse T3, if available), developmental delay (all unless otherwise explained) (**Recommendation: S,Quality of evidence:** ØØOO).
- We **suggest** that *THRA* sequencing be **considered** in any patient with:
  two major criteria, as listed above (**Recommendation: W, Quality of evidence:** ØØOO)

- one major criterion, as listed above, plus two or more of the following minor criteria: unexplained anemia, clinical features of hypothyroidism (dysmorphic facies), neurocognitive features (dyspraxia), skeletal dysplasia (**Recommendation: W, Quality of evidence:** ØØOO)
- three or more minor criteria, as listed above. (**Recommendation:** **W, Quality of evidence:** ØØOO)

- We **suggest** that *THRA* sequencing be performed even if the criteria above are not met but clinical suspicion of RTHα remains (**Recommendation: W, Quality of evidence:** ØØOO).
- If a variant of uncertain significance (VUS) in *THRA* is identified, we **suggest**that the following methods can be used to determine whether it is pathogenic, recognizing that such evaluation will require liaison with clinical genetics services or international laboratories/clinical services with expertise in the diagnosis and/or management of RTHα:
  - Determination of variant genotype–phenotype segregation in families
  - Assessment of whether there is a *THRB* mutation known to cause RTHβ homologous with the *THRA* variant in the patient.
  - Structural modelling of the variant in TRα and assessment whether the change in protein structure is predicted to affect the normal function of TRα
  - Testing the TRα variant in assays of receptor function
**(Recommendation: W, Quality of evidence: ØØOO)**

- We **do not recommend**the sole use of predictive algorithms (e.g. PolyPhen, SIFT, REVEL, CADD) to determine the pathogenicity of a VUS in *THRA* (**Recommendation: S, Quality of evidence:** ØØOO).
- As the phenotypic spectrum of this disorder is not fully defined, we **do not recommend** making a definitive diagnosis of RTHα in individuals who do not have a pathogenic *THRA* mutation(**Recommendation: S, Quality of evidence:** ØØOO).

*Management and treatment*

- Following the diagnosis of RTHα, we **recommend** a trial of levothyroxine therapy in all patients (**Recommendation: S, Quality of evidence:** ØØOO).
- We **recommend** the continuation of levothyroxine therapy long-term in all patients unless concerns of side effects or tolerability arise (**Recommendation: W, Quality of evidence:**ØOOO).
- We **suggest** all patients with RTHα remain under the care of an endocrinologist lifelong (**Recommendation: S, Quality of evidence:**ØOOO).
- We **suggest** referral to other specialities, as necessary, including neurology, neuropsychology, gastroenterology, hematology, dental services, physiotherapy, speech and language therapy, and occupational therapy (**Recommendation: S, Quality of Evidence: Quality of evidence:**ØOOO).
- In children with RTHα and impaired GH secretion or low baseline IGF-1 concentrations, we **recommend** reassessment of GH status after levothyroxine therapy (**Recommendation: W, Quality of evidence:** ØØOO).
- There is insufficient data to provide recommendations on the dosage of levothyroxine (though it should be above the usual replacement dose), or to specify clinical or biochemical targets of therapy (**Recommendation: W, Quality of evidence:**ØOOO).
- There is insufficient data to recommend for or against the use of liothyronine in RTHα at present (**Recommendation: W, Quality of evidence:**ØOOO).
- There is insufficient data to provide recommendations on the management of RTHα during pregnancy; however, we **suggest** continuation of levothyroxine therapy during pregnancy (**Recommendation: W, Quality of evidence:**ØOOO).
- We **recommend** against the use of liothyronine during pregnancy(**Recommendation: S, Quality of evidence:**ØOOO).

**Box 3**. Summary of key recommendations - MCT8 deficiency.

*Diagnosis*

- When considering a diagnosis of MCT8 deficiency, we **recommend** a full clinical assessment to determine whether the patient exhibits any recognized clinical phenotypes and a physical examination, including neurological assessment (**Recommendation: S, Quality of evidence:** ØØOO).
- If MCT8 deficiency is suspected, we **recommend** additional investigations including (but not limited to) serum (free) T3, (free) T4, reverse T3 (rT3) and TSH concentrations (interpreting results in the context of age-specific reference intervals), (**Recommendation: S, Quality of evidence:** ØØOO**)**.
- We **recommend** that *SLC16A2* sequencing be **performed** in any male patient with:
  - the following ‘major criteria’; typical biochemical profile (TSH within normal range or mildly raised, low or low-normal (F)T4, raised (F)T3, low reverse T3, and/or elevated (F)T3/(F)T4 or T3/rT3 ratio) in combination with either global developmental delay, hypomyelination on MRI, clinical signs of movement disorder (e.g. dystonia, bradykinesia), persistent primitive reflexes, or a positive family history for MCT8 deficiency (**Recommendation: S, Quality of evidence:** ØØOO).
- We **suggest** that *SLC16A2* sequencing be **considered** in any patient with:
  ii. characteristic thyroid function tests (TSH within normal range or mildly raised, low or low-normal T4, raised T3, low reverse T3, and/or elevated T3/(F)T4 or T3/rT3 ratio) and subtle developmental delay or behavioral abnormalities (**Recommendation: W, Quality of evidence:**ØOOO).
- We **suggest** that *SLC16A2* sequencing be **considered** prenatally (through villus sampling or amniocentesis) in pregnancies with male fetal sex if the family history is positive for MCT8 deficiency and genetic segregation indicates a risk of the fetus carrying the mutant allele (**Recommendation: W, Quality of evidence:** ØOOO).
- If a variant of uncertain significance (VUS) in *SLC16A2* is identified, we **recommend** using the following methods to determine pathogenicity:
  - segregation of genotype with phenotype in families.
  - testing the function of novel variants in transfected cells or patient-derived cells
  - structural modeling.
    (**Recommendation: W, Quality of evidence:** ØOOO)

- We **do not recommend**the sole use of predictive algorithms (e.g. PolyPhen, SIFT, REVEL, CADD) to determine the pathogenicity of a VUS (**Recommendation: S, Quality of evidence:** ØOOO).
- The pathways described above may require liaison with clinical genetics services or international laboratories/clinical services with expertise in the diagnosis of MCT8 deficiency.

*Management and treatment*

- Post-natal treatment with levothyroxine monotherapy is **not recommended** (**Recommendation: S, Quality of evidence:** ØØOO).
- We **recommend**treatment with TRIAC (**Recommendation: S, Quality of evidence:** ØØØO) or DITPA (**Recommendation: W, Quality of evidence:** ØØOO).
- We **recommend** titrating the dosage of TRIAC (or DITPA), aiming to reduce serum T3 concentrations to a target range between 1.4 and 2.5 nmol/L to alleviate the peripheral thyrotoxic state, unless limited by the occurrence of dose-related side effects (**Recommendation: S, Quality of evidence:** ØØØO). A higher dosage of TRIAC can reduce serum T3 concentrations to the lower end or below the age-specific reference range, potentially benefiting neurodevelopment (**Recommendation: W, Quality of evidence:**ØOOO).
- We **recommend** long-term therapy with TRIAC (or DITPA) in all patients unless concerns about drug side effects or tolerability arise (**Recommendation: W, Quality of evidence:** ØØOO).
- If thyroid hormone analogs are unavailable, we **suggest**that a combination of levothyroxine and PTU treatment could be considered (**Recommendation: W, Quality of evidence:** ØØOO).
- Given the rare but potentially severe and life-threatening side effects of PTU and the unknown long-term effects of thyroid hormone analogs, we acknowledge that the risks versus benefits of such therapies should be considered carefully, particularly in patients whose baseline liver function is already deranged (1) (**Recommendation: S, Quality of evidence:** ØØOO).
- We **recommend**all patients with MCT8 deficiency remain under the care of a (pediatric) endocrinologist and a (pediatric) neurologist (**Recommendation: S, Quality of evidence:**ØOOO).
- We **recommend**the discussion of cases by a multidisciplinary team, comprising (pediatric) endocrinologists, neurologists, cardiologists, dietitians, occupational, speech and physiotherapists, physicians in rehabilitation medicine (physiatrists), orthopedic surgeons, and medical daycare centers (**Recommendation: S**); oversight of multidisciplinary team outcomes by a case manager is valuable (**Recommendation: S, Quality of evidence:** ØOOO).
- We **recommend** careful evaluation of dietary intake to maintain body weight that is adequate for age, and addressing feeding problems to prevent undernutrition. We **suggest**initiating percutaneous enteral tube feeding and the input of a dietitian to ensure adequate caloric intake at an early stage to prevent malnutrition (**Recommendation: S, Quality of evidence:** ØØOO).
- We **suggest**that all patients should be offered empirical symptomatic treatment for neurological symptoms, including dystonia/hypertonia (spasmolytic drugs), drooling (e.g. anticholinergic drugs), and true epilepsy (carefully distinguished from a paroxysmal movement disorder); and should be referred to rehabilitation physicians/orthopedic surgeons/physiotherapists for measures anticipating contractures, scoliosis, a hip subluxation (**Recommendation: S, Quality of evidence:** ØØOO).
- We **suggest** treating frequently occurring gastrointestinal issues such as gastroesophageal reflux and/or gastroparesis, as well as constipation in accordance with standard practice (**Recommendation: S, Quality of evidence:** ØØOO).

**Box 4.** Summary of key recommendations – Disorders of thyroid hormone metabolism.

**Recommendations in selenoprotein deficiency**

*Diagnosis*

- We **recommend** measurements to identify raised serum FT4, normal or low FT3, raised reverse T3, and low plasma selenium concentrations (**Recommendation: S, Quality of evidence:** ØØOO).
- When making a genetic diagnosis, we **recommend** next-generation (whole exome or genome) sequencing of *SECISBP2*, enabling identification of intronic mutations as recorded in several cases. In cases of proven selenoprotein deficiency with an apparent, monoallelic, gene defect, we **recommend** analysis of SECISBP2 cDNA to identify missplicing events due to cryptic intronic mutations involving the other allele. Reduced expression or function of selenoproteins in patient-derived cells is also informative (**Recommendation: S, Quality of evidence:** ØØOO).

*Management*

- We **recommend** monitoring for growth retardation and delayed development in childhood (**Recommendation: S, Quality of evidence:** ØØOO).
- We **recommend** periodic magnetic resonance imaging and **suggest** muscle biopsy in selected cases to identify and monitor the evolution of muscular dystrophy, with measurements of vital capacity and polysomnography to detect nocturnal hypoventilation (**Recommendation: S, Quality of evidence:** ØØOO).
- We **recommend** surveillance of patients for progressive, sensorineural hearing loss and aneurysmal dilatation of the thoracic aorta (**Recommendation: S, Quality of evidence:** ØØOO).
- We **suggest** monitoring patients for cutaneous photosensitivity, and male infertility (**Recommendation: W, Quality of evidence:** ØØOO).

*Treatment*

- We **recommend** liothyronine therapy in patients with subnormal FT3 concentrations (**Recommendation: S, Quality of evidence:** ØØOO).
- We **do not recommend** selenium supplementation in SECISBP2 deficiency (**Recommendation: S, Quality of evidence:** ØØOO).
- We **suggest** that antioxidant (e.g. alpha-tocopherol) treatment to prevent oxidative stress-induced tissue damage can be considered (**Recommendation: W, Quality of evidence:** ØØOO).

**Recommendations in iodothyronine deiodinase defects**

*Diagnosis*

- When considering a diagnosis of a D1 defect, we **recommend** first documenting raised serum rT3 concentrations and an elevated rT3:T3 ratio in the absence of non-thyroidal illness (see Supplementary figure 3) (**Recommendation: W, Quality of evidence:** ØOOO).
- As inheritance is dominant, testing other family members, in particular the parents, is very helpful (**Recommendation: S, Quality of evidence:** ØØOO).
- As with other disorders, such as the *SECISBP2* mutation, which can exhibit the same thyroid hormone abnormalities, demonstrating a mutation in the *DIO1* gene is paramount (**Recommendation: S, Quality of evidence:** ØØOO).
- New *DIO1* mutations would require demonstration of a functional abnormality (**Recommendation: W, Quality of evidence:**ØOOO).
- There are no other reported clinical or biochemical manifestations, but the loss of D1 function can alter the thyroid tests associated with other thyroid defects (**Recommendation: W, Quality of evidence:**ØOOO).

## Differential diagnosis of raised thyroid hormones and non-suppressed TSH

**Figure 1:**
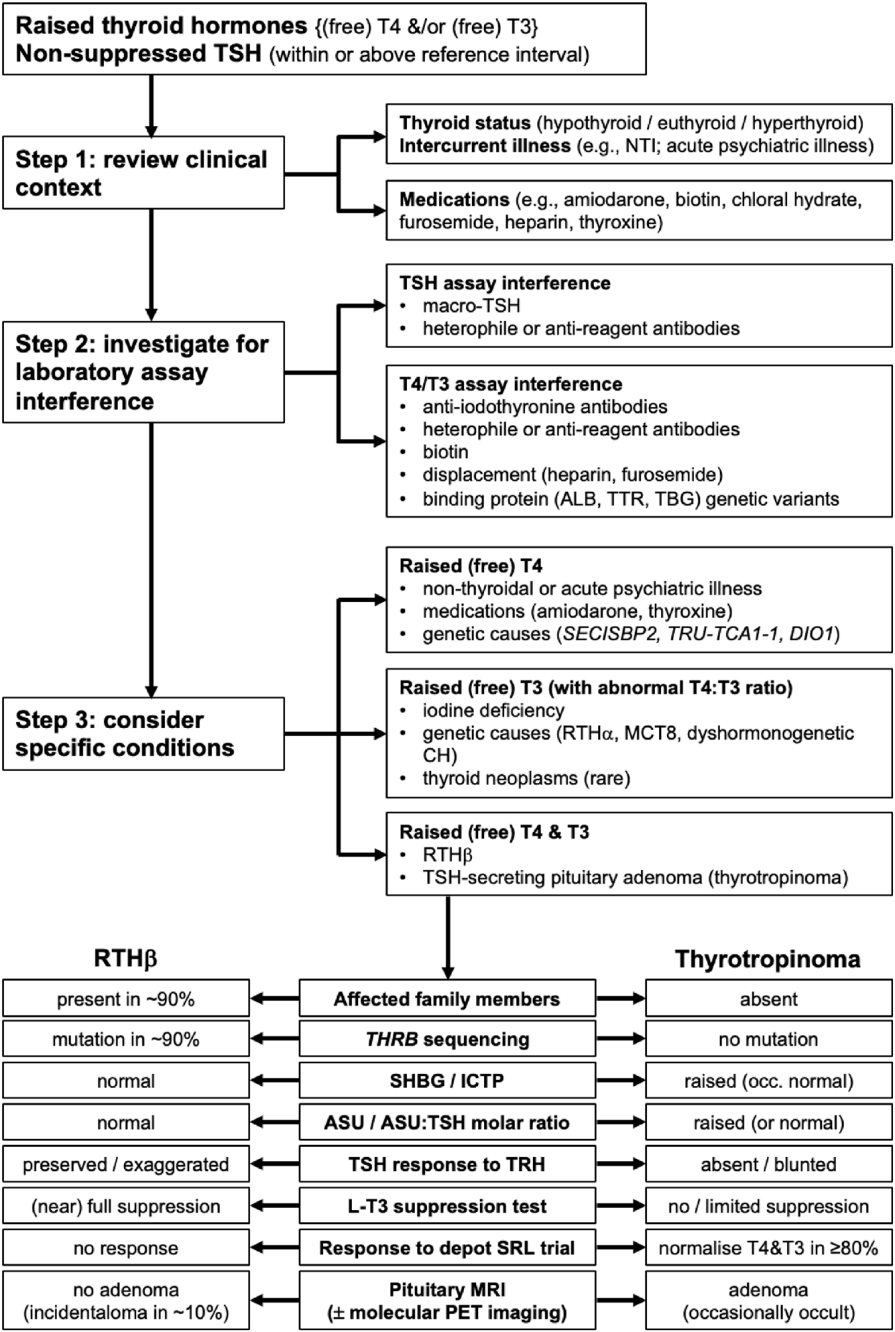
Algorithm for differential diagnosis, showing stepwise approach to the investigation of raised thyroid hormones (T4 and/or T3) with non-suppressed TSH. Key: ALB, albumin; ASU, alpha subunit; CH, congenital hypothyroidism; DIO1, deiodinase type 1; ICTP, serum carboxy-terminal telopeptide of type 1 collagen; L-T3, liothyronine; MCT8, monocarboxylate transporter 8; MRI, magnetic resonance imaging; NTI, non-thyroidal illness; PET, positron emission tomography; RTHα, resistance to thyroid hormone alpha; RTHβ, resistance to thyroid hormone beta; SECISBP2, selenocysteine insertion sequence binding protein 2; SHBG, sex hormone binding globulin; SRL, somatostatin receptor ligand; T3, triiodothyronine; T4, thyroxine; TBG, thyroxine binding globulin; THRB, thyroid hormone receptor beta gene; TRH, thyrotropin releasing hormone; TRU-TCA1-1, tRNA selenocysteine (anticodon TCA) 1-1; TSH, thyrotropin; TTR, transthyretin.

The finding of thyroid hormones {(free) T4 and/or (free) T3} above the reference interval with non-suppressed thyrotropin (TSH) can be one of the most challenging patterns of discordant thyroid function tests (TFTs) to resolve. A systematic approach (Fig.1) is required to prevent inappropriate further investigations and unnecessary treatments, while at the same time ensuring rare genetic and acquired disorders are diagnosed in a timely manner.

A key first step in the evaluation of a patient with hyperthyroxinemia and non-suppressed TSH is to exclude potential confounding factors (including intercurrent non-thyroidal physical or psychiatric illness) and medications (e.g. amiodarone, thyroxine). At the same time, the patient’s clinical thyroid status should be determined (Fig. 1, Step 1) (6, 7).

In the absence of a readily explainable physical or pharmacological cause for the abnormal TFTs, close cooperation with the clinical biochemistry laboratory is necessary to investigate possible interference in one or more assays (Fig. 1, Step 2). Here, information from the clinical assessment helps guide the approach to excluding artefactual elevation in T4, T3, or TSH (e.g. in a patient with clinical features of hyperthyroidism, initial suspicion should focus on the reliability of TSH measurement). The presence of interfering antibodies (including both analyte-specific (e.g. macro-TSH, anti-iodothyronine) and those targeting reagents in commonly used immunoassays (e.g. heterophile or anti-animal immunoglobulins)) requires specific exclusion. Demonstration of discrepant findings using different assay platforms (method comparison) is strongly suggestive of interference, but additional steps may be required for detection/confirmation (e.g. dilution studies, polyethylene glycol (PEG) precipitation, or gel filtration chromatography for suspected TSH interference; equilibrium dialysis or ultrafiltration for suspected T4 or T3 interference) (7, 8).

Once laboratory assay artefact has been excluded, and genuine hyperthyroxinemia with non-suppressed TSH is confirmed, judicious use of additional tests (including (free) T3 if not already measured (with calculation of the T4:T3 ratio), reverse T3 (rT3), thyroid autoantibodies, sex hormone-binding globulin (SHBG)) can provide important clues to the underlying diagnosis (Table 1) and guide the next phase of investigation (Fig. 1, Step 3) (7).

**Table 1:** Key biochemical and clinical features in genetic disorders of thyroid hormone transport, metabolism, and action.

	Disorder				
	RTHβ	RTHα	MCT8 deficiency	SP deficiency	DIO1 deficiency
Gene	*THRB*	*THRA*	*SLC16A2*	*SECISBP2 or TRU-TCA 1-1*	*DIO1*
Biochemical signature					
Free T4	High	Low-normal or low	Low-normal or low	High	Normal or slightly high
Free T3	High	High-normal or high	Usually high or high-normal	Low or normal	Normal
Reverse T3	High	Normal or low	Low	High	High
TSH	Normal or high	Normal (or mildly raised)	Normal (or mildly raised)	Normal	High
SHBG	Normal	Normal or high	High	High	–

DIO1, deiodinase type 1; MCT8, monocarboxylate transporter 8; RTHβ, resistance to thyroid hormone beta; RTHα, resistance to thyroid hormone alpha; SP, selenoprotein.

Distinction between resistance to thyroid hormone beta (RTHβ) and TSH-secreting pituitary adenoma (thyrotropinoma or TSHoma) typically requires a multimodal approach, including biochemical investigation, genetic screening, dynamic endocrine testing, pituitary imaging, and a possible trial of depot somatostatin receptor ligand (SRL) therapy (Fig. 1, Step 3) (9, 10).

## Resistance to thyroid hormone β

### Diagnosis

- The key characteristic of resistance to thyroid hormone beta (RTHβ) (OMIM 188570: Thyroid hormone resistance, generalized; OMIM 145650: Thyroid hormone resistance, pituitary; ORPHA: 566243) is a combination of genuinely raised (total and free) thyroid hormones (T4, T3), with non-suppressed TSH (11, 12, 13, 14)). The diagnosis is also suspected based on other phenotypes described below (and summarized in Table 2).

**Table 2:** Clinical features and investigations in resistance to thyroid hormone β.

System	Clinical features	Investigation^a^
Thyroid	Goiter, nodules, thyroid cancer, thyroid autoimmunity	**Ultrasound scan of thyroid; FNA cytology of radiologically indeterminate/suspicious nodules; thyroid autoantibodies**
Metabolic	Abnormal thyroid function, failure to thrive; low body mass index; dyslipidemia, MASLD, insulin resistance	**TSH, Free T4, Free T3** (reverse T3, TBG); resting energy expenditure; muscle creatine kinase, SHBG, angiotensin-converting enzyme; **fasting lipid profile**;fibroscan; **fastingglucose,** HbA1c, insulin
Skeletal	Growth retardation, osteopenia/osteoporosis, stippled epiphyses (biallelic cases)	Pelvis and long bone radiographs, spine radiograph: vertebral fractures; **DXA**: reduced bone mineral density (hip, spine); **calcium**, **25OH-vitamin D**, **PTH;**Markers of bone formation or resorption: P1NP/CTX/BAP (osteocalcin/NTX) (unlike conventional thyrotoxicosis, bone turnover markers are usually normal)
Audiovisual function	Recurrent ENT infections; hearing loss; altered color vision sensitivity	Audiometry; AABR tests; Farnsworth–Munsell 100 Hue test with calculation of total error score; light- and darkness-adapted electroretinograms (ERG), with cone-specific chromatic stimuli.
Neurological and cognitive	Peripheral tremor, ADHD, emotional disturbance (anxiety; insomnia), learning disabilities/memory loss; mental retardation (especially homozygous cases)	**Neuropsychological testing for ADHD** (Rating Scale-IV or Conners Rating Scales) and other cognitive deficits; Wechsler IQ scale.
Appearance	No typical facial features or body habitus (except goiter)	–
Pregnancy	Increased miscarriage rate in the first trimester; LBW/SGA (unaffected babies)	**Expert ultrasound monitoring of fetal growth and development** (as for follow-up of Graves’ disease), Monitoring of maternal thyroid hormones
Cardiovascular	Tachycardia, atrial fibrillation, cardiac insufficiency	**Resting ECG**; **cardiactelemetry**;echocardiography: hyperthyroid indices of cardiac contractility; BNP or NT-pro-BNP

AABR, automated auditory brainstem response; ADHD, attention deficit hyperactive disorder.

^a^**Key investigations are in bold.**
- After excluding assay interference or other causes of this abnormal hormone pattern, distinguishing between RTHβ or a pituitary TSH-secreting adenoma (TSHoma) is made based on clinical, dynamic endocrine, and radiological investigations outlined above.
- Heterozygous pathogenic variants in *THRB* are identified by conventional (Sanger) gene sequencing (which may not be available in resource-limited settings) in most suspected RTHβ cases and confirm the diagnosis (Supplementary Figure 1, see section on supplementary materials given at the end of this article), but in 10% of individuals with this clinical and biochemical phenotype, a *THRB* defect cannot be identified. Somatic mosaicism for a *THRB* variant that is not expressed in all tissues may account for a subset of such negative cases, and next-generation sequencing at higher read depth may enable the diagnosis of RTHβ in this context (15, 16).
- Consistent with the autosomal dominant inheritance of RTHβ, mutant TRβ inhibits the function of its normal (or wild-type) receptor counterpart in a dominant negative manner (17). Receptor loss-of-function is usually due to reduced hormone binding affinity (18, 19), but some TRβ mutants exhibit impaired corepressor dissociation (20) or coactivator recruitment (21, 22, 23).
- When treating autoimmune hypothyroidism (24, 25, 26, 27) or congenital hypothyroidism (28, 29, 30, 31), the inability of thyroxine replacement (even in supraphysiological dosages) to normalize circulating TSH can suggest underlying coexistent RTHβ.
- Hyperthyroidism (due to Graves’ disease, thyroiditis, or amiodarone-induced) can mask underlying RTHβ, which is suspected when antithyroid drug or other treatment results in a marked or exaggerated rise in TSH concentrations in the face of normal circulating thyroid hormones (25, 32, 33).

### Clinical features

RTHβ patients can exhibit features of hypothyroidism or hyperthyroidism, reflecting either hormone resistance in TRβ-expressing tissues or approximately normal sensitivity to elevated circulating thyroid hormones in TRα-expressing tissues.

### Thyroid

- A goiter, with eventual nodular change, can often be present in RTHβ (34), possibly due to enhanced bioactivity of circulating TSH in this disorder (35).
- Rarely, cases of RTHβ with thyroid cancer (generally papillary microcarcinoma; one metastatic) have been reported (36, 37, 38, 39) with favorable clinical outcomes despite incomplete TSH suppression following thyroid ablation (38).
- Patients with RTHβ have a higher risk of developing thyroid autoimmunity (40).

### Cardiovascular phenotype

- Hyperthyroid cardiac manifestations, mediated by the action of elevated thyroid hormones on TRα-expressing myocardium, include tachycardia/tachyarrhythmias (in most cases), atrial fibrillation, and cardiac insufficiency (41, 42, 43, 44). RTHβ patients are at significantly higher risk of adverse cardiovascular outcomes (atrial fibrillation, myocardial infarction, heart failure) and of earlier mortality (45).

### Metabolism

- Resistance to hormone action in TRβ-expressing liver likely mediates normal serum SHBG (46, 47), mixed dyslipidemia (raised cholesterol, triglyceride), and increased liver fat (MASLD) in RTHβ patients (48, 49, 50, 51). The respective roles of muscle or liver insulin resistance in the metabolic picture are not clearly delineated. Similarly, whether adverse cardiovascular outcomes are due to dyslipidemia and insulin resistance causing increased atherosclerosis or represent a direct, thyrotoxic cardiomyopathy or a combination remains unknown.

### Neurological and audiovisual phenotypes

- Neurocognitive manifestations of RTHβ include anxiety and sleep disturbance, attention deficit hyperactivity disorder (52, 53), mild intellectual disability (lower nonverbal intelligence), language difficulties (54), and poorer academic outcomes (55). Severe intellectual disability in homozygous cases (56) suggests that either mutant TRβ can interfere with the function of TRα1 or a role for TRβ pathways in brain development.
- Hearing loss in RTHβ is due to a combination of conductive deficit (secondary to frequent ear infections in childhood) and cochlear dysfunction (34, 57).
- Impaired color perception is present in heterozygous RTHβ patients (58) with frankly abnormal color vision only in rare homozygous cases (59, 60). Macular dystrophies have been recorded in patients with a *THRB* splice variant (61).

## Fertility and pregnancy

- An increased rate of miscarriage has been reported in maternal RTHβ patients (62, 63). Following exposure to high maternal TH during gestation, unaffected infants born to RTHβ females are small for gestational age, have a suppressed TSH, and exhibit low birth weight (62, 63, 64, 65).

### Genotype-phenotype correlation

- The clinical phenotype of RTHβ is highly variable, ranging from asymptomatic individuals to patients with thyrotoxic features (66). The magnitude of elevation in circulating free T4 (67), resting energy expenditure (68), or LDL cholesterol (50) correlates with the *THRB* genotype, for a subset (so-called type 1) of TRβ mutations whose loss-of-function is proportional to their degree of impairment in hormone binding (67). Rare cases with homozygous deletion (59) or variants (56, 69) in *THRB* exhibit features including dysmorphic facies and audiovisual abnormalities (59) or intellectual disability, tachyarrhythmias, and thyrotoxic cardiomyopathy (56, 69, 70).

## Treatment of RTHβ

- Case reports and small case series have shown that TRIAC (triiodothyroacetic acid), a central thyromimetic which inhibits TSH secretion to lower circulating TH, when used alone or in combination with beta blockade, controls thyrotoxic signs and symptoms in RTHβ, both in adults (71) and children (72, 73). A dosage of 1.4 to 2.8 mg administered twice or three times daily is most effective (74), in keeping with its half-life (75), and ameliorates ADHD symptoms (76, 77). The combination of antithyroid drug and TRIAC can control thyrotoxic cardiomyopathy without a rise in TSH and goitrogenesis (70). Although not yet licensed for this indication, TRIAC can be prescribed in individual RTHβ cases via the manufacturer’s managed access programs or via Galenic formulations of the drug made by pharmacists.
- Alternate day T3 administration was reported to reduce large goiter volume in one RTHβ patient (78). Liothyronine (but not methylphenidate) therapy ameliorated ADHD symptoms (79).
- Coexistent RTHβ complicates the treatment of hypothyroidism, with thyroid hormone replacement needing to balance restoration of normal thyroid status with avoidance of tissue (e.g. cardiac) hyperthyroidism (80). Chronic underreplacement with levothyroxine in hypothyroid RTHβ cases risks the development of pituitary thyrotroph hyperplasia (81).

### Areas of uncertainty

- Whether prenatal diagnosis or antithyroid drug therapy to alter maternal TH concentrations in an RTHβ pregnancy is warranted.
- Whether neonatal diagnosis and early intervention can improve neurological or behavioral phenotypes of RTHβ.
- Whether lipid-lowering or TRIAC therapy, alone or in combination, can alter adverse cardiovascular outcomes in this disorder.
- Whether low bone density in RTHβ increases fracture risk; do antiresorptive or other therapies prevent bone loss or affect fracture risk?
- Further evaluation of therapies (e.g. TRIAC, liothyronine) for ADHD or other neuropsychological phenotypes is warranted.
- Whether the disorder can be caused by heterozygous (or homozygous) variants in *THRB*, outside the recognized mutation hotspots or clusters in its hormone-binding domain.
- Whether genome-wide sequencing should be considered in suspected RTHβ cases without a germline or somatic mosaic mutation in TRβ, looking for either an abnormality in the non-coding region of *THRB* or a defect in an unrelated gene, causing this phenotype.

## Resistance to thyroid hormone α

### Clinical features and making a diagnosis

Resistance to thyroid hormone α (OMIM 614450: Congenital Nongoitrous Hypothyroidism 6; ORPHA: 566231) is a rare disorder, with 41 affected individuals reported to date (Supplementary Table 1) (82, 83, 84, 85, 86, 87, 88, 89, 90, 91, 92, 93, 94, 95, 96, 97, 98, 99, 100). Although the phenotype is highly variable, many patients exhibit similar clinical features, summarized in Table 3. As knowledge of the phenotypic spectrum of RTHα is changing and will probably expand in the future, it is not possible to provide definite criteria that warrant *THRA* sequencing to make a diagnosis. However, to aid physicians’ decision-making when considering this diagnosis, we propose using criteria (which may evolve as more cases are described) to direct further investigation. A definitive diagnosis of RTHα is made following the identification of a pathogenic mutation in *THRA.* We acknowledge that *THRA* sequencing is not available in all countries and also that some cases of RTHα have been described in whom *THRA* mutations have not been identified (87).

**Table 3:** Clinical features and investigations in resistance to thyroid hormone α.

System	Clinical feature/phenotype	Investigation^a^
Appearance	Flattened nasal bridge, broad face, thickened lips, macroglossia, coarse facies; skin tags and moles	Photographs
Neurological and cognitive	Delayed childhood milestones; slow speech and initiation of movement; ataxic gait; dysdiadochokinesis; fine and gross motor incoordination (dyspraxia)	MRI scan: .cortical and cerebellar involution; Wechsler IQ scale: reduced perceptual reasoning, processing speed, and visuomotor integration
Skeletal	Growth delay; short stature (often disproportionate); macrocephaly: increased head circumference (SDS or centile); delayed tooth eruption	**Growth chart:** reduced total height; normal sitting height (upper segment); reduced subischial leg length.
		**Head circumference:** increased (Centile chart or SDS); Skull radiograph; thickened calvarium; delayed fontanelle fusion; excessively serpiginous lambdoid suture (wormian bones); pelvis and long bone radiographs; femoral epiphyseal dysgenesis, cortical hyperostosis; spine radiograph: scalloped vertebral bodies; dental radiograph: delayed tooth eruption; wrist radiograph: delayed carpal bone maturation (bone age)
Gastrointestinal	Constipation	Abdominal radiograph: dilated bowel loops, fecal impaction; measurement of colonic transit time using radio-opaque markers or other locally available investigations; colonic manometry: reduced peristalsis frequency
Cardiovascular	Bradycardia (mild); low BP for age/gender; pericardial effusion (n2)	Cardiac telemetry (average sleeping heart rate); echocardiography: high pre-ejection period, low cardiac index, low E/A ratio, low LV ejection fraction; pericardial effusion; spectral analysis of cardiac autonomic tone^b^
Metabolic	Low metabolic rate; thyroid function tests often borderline abnormal (but can be normal)	**Muscle creatine kinase:** often raised; **SHBG:**often raised; **TSH:** within reference range or borderline elevated; **Free T4/Free T3 ratio**:often low Reverse T3: often low; **serum IGF-1:**may be low; DXA: body composition (fat mass and fat-free (lean) mass^b^; indirect calorimetry: resting energy expenditure^b^
Hematological	Mild anaemia	**Full blood count:** red cell mass and hematocrit can be low; hematinics (iron, B12, folate, hemolytic indices, EPO concentrations) usually normal

^a^Key investigations are in bold; ^b^Indicates investigations that are often only available on a research basis.

### Management and treatment

- To date, thyroid hormone therapy (almost always levothyroxine, with liothyronine only used in one case (89)), is the only treatment described for RTHα. Although data are restricted to case reports or case series and therapeutic responses are variable, thyroxine treatment in RTHα seems safe and well tolerated and provides beneficial effects for most patients.
- The dosage of levothyroxine used varies (up to 3.8 mcg/kg/day), with serum TSH remaining normal in some T4-treated patients (95, 101), but suppressed TSH with elevated TH concentrations being recorded in other cases (82, 84, 85, 86, 90, 93, 97, 98, 101).
- Levothyroxine therapy has proven beneficial for constipation (82, 84, 86, 93, 95, 98, 99, 101) and growth (82, 84, 90, 93). Cardiac responses to therapy are also mixed, with improvement in contractile function (85, 86) and a rise in heart rate (84, 85), without resultant tachycardia (84, 85, 102). Anaemia, if present, has consistently shown little or no change following levothyroxine (85, 86, 93, 98, 101). Change in neurocognitive function is variable, with improved emotional affect reported in some patients (86, 90, 93, 97, 101), but no benefit in other cases (84, 99). Where tested, nerve conduction improved in a single case (84). Low baseline IGF-1 concentrations in many children with RTHα may (82, 84), or may not (101) normalize following levothyroxine therapy. GH response to provocation can be normal (82) or subnormal (84).
- It is recognized that levothyroxine therapy, in dosages causing suppressed TSH and elevated TH concentrations, may be required to overcome hormone resistance in TRα-expressing tissues, with the potential for thyrotoxicosis in TRβ-expressing tissues (103). Overall, it is likely that an individual RTHα patient’s response to levothyroxine therapy depends on the severity of the underlying receptor defect, timing and dosage of drug therapy, and TH concentrations achieved (93).

### Areas of uncertainty

- Optimal biomarkers to diagnose RTHα and assess its response to levothyroxine therapy are undefined.
- Appropriate treatment targets and whether these should be different in childhood (e.g. linear growth, neurodevelopmental outcome) versus adult life (hypothyroid symptoms) are uncertain.
- Whether levothyroxine therapy, in apparent supraphysiological dosage, is associated with tissue thyrotoxicosis and adverse outcomes is unknown.
- There is no information to guide the management of maternal RTHα prior to conception or during pregnancy.
- Whether the diagnosis of the disorder and commencement of levothyroxine therapy at birth can prevent adverse neurodevelopmental outcomes is unknown.

## Monocarboxylate transporter 8 deficiency

### Clinical features

MCT8 deficiency (OMIM 300523: Allan–Herndon–Dudley syndrome or MCT8 deficiency; ORPHA:59) is characterized by a varying neurodevelopmental delay due to cerebral hypothyroidism, and a wide range of clinical sequelae secondary to chronic peripheral tissue thyrotoxicosis caused by elevated serum T3 concentrations (104) (summarized in Table 4).

**Table 4:** Clinical phenotype and investigation of MCT8 deficiency^a^.

System	Clinical feature/phenotype	Investigation^b^
Appearance	Myopathic facies, dystrophic	Photograph
Neurological and cognitive	Severely delayed cognitive and motor development; central hypotonia (e.g. poor head control); dystonia; spasticity (later in life); persistent primitive reflexes; seizures	**Neuropsychological tests** (Bayley scales of infant and toddler development; gross motor function measure); MRI scan: can show hypomyelination; EEG
Endocrine	Thyrotoxic features (increased perspiration, tachycardia, low body weight)	**TSH, (F)T4,(F)T3,reverseT3FreeT4/freeT3ratio**: often low; serum markers of thyroid status^c^: SHBG (n/↑), CK (n/↓), creatinine (n/↓), total cholesterol (n/↓), ALAT (n/↑)
Skeletal	Scoliosis;hip subluxation;osteoporosis	Radiographs of spine and hip; DEXA
Cardiovascular	Tachycardia, conduction abnormalities; systolic hypertension	**ECG**; Cardiac telemetry; Blood pressure
General	Low body weight; gastroesophageal reflux; feeding problems; constipation	**Nutritional assessment by dietitian; measure body weight every 3 months in infants and children**

^a^An X-linked disorder usually affecting male individuals; ^b^Key investigations are in bold; ^c^For recommended serum measurements, changes typically observed in individuals with MCT8 deficiency are indicated between parentheses.

↑ raised, ↑↑ very raised, ↓ low, ↓↓ very low, n, normal; TFTs, thyroid function tests.

### Making a diagnosis

- Individuals with MCT8 deficiency have been identified by targeted sequencing of *SLC16A2,* on the X chromosome, in selected individuals (usually male) with clinical and biochemical characteristics, or by exome sequencing strategies (including specific gene panels, e.g. global developmental delay, hypotonia, spasticity, and seizures).
- Definitive diagnosis of MCT8 deficiency requires identification of a known pathogenic mutation, either by reliable *in silico* prediction and/or functional studies of novel variants in transfected cells or patient-derived cells.
- Although the presence and severity of disease features in individuals with mutations in *SLC16A2* can vary, several core characteristics (Table 4) are consistently present and necessitate *SLC16A2* sequencing.

## Management and treatment

- Treatment of MCT8 deficiency should ideally aim to
  - increase thyroid hormone action in the hypothyroid brain.
  - ii. ameliorate the thyrotoxic state of peripheral tissues.
- Thus far, treatments with levothyroxine (alone or in combination with propylthiouracil (PTU)), and the T3 analogues diiodothyropropionic acid (DITPA) and triiodothyroacetic acid (TRIAC) have been described.
- Knowledge of response to thyroxine (alone, or in combination with PTU) and DITPA is limited to case reports and case series, whereas the effects of TRIAC have been studied in a phase 2 clinical trial and a prospective cohort study.
- Treatment with levothyroxine in a wide dose range (2.5–15 mcg/kg/day), commenced at the age of 0.5 to 36 months, did not improve neurodevelopment and, in some cases even aggravated the hyperthyroid state in peripheral tissues (105, 106, 107, 108, 109, 110, 111, 112, 113, 114, 115, 116, 117, 118).
- Combination therapy with propylthiouracil (PTU− given to block T4 to T3 conversion) and levothyroxine reduced signs of hyperthyroidism in peripheral tissues in some patients, increasing body weight, reducing heart rate, and serum sex hormone-binding globulin (SHBG) concentrations, but had no beneficial effects on neurocognitive development (119, 120, 121, 122).
- Treatment with DITPA (dose range: 2.1–2.5 mg/kg/day) normalized serum T3 and TSH without reduction of T4 concentrations (4/4 cases) and had mixed effects on peripheral symptoms, including a reduction in heart rate (3/4 cases) and improvement in body weight (2/4 cases). Commencement of treatment between 8.5 and 25 months did not improve neurocognitive development (122).
- TRIAC therapy (dose range: 6.4–84.3 mcg/kg/day) has proven to have beneficial effects for some features:
  - Increases body weight (123, 124).
  - Cardiovascular endpoints, including lower resting heart rate, number of premature atrial contractions, and systolic blood pressure (124).
  - Decreases serum T3 concentrations and markers of thyroid hormone status in peripheral tissues (e.g. SHBG) (123, 124).

A formulation of TRIAC (Emcitate®) that has been trialed in MCT8 deficiency is available in several countries; in other countries, Galenic formulations of the drug made by pharmacists may be available.

- The neurocognitive response to TRIAC treatment is unknown (123, 124), and is currently under investigation (NCT02396459).
- Appropriate treatment targets may vary with age. For example, improved neurodevelopmental outcomes may be the most important goal in neonates but not adults; however, gaining body weight is beneficial to patients of all ages. Attaining these treatment targets may require different therapeutic strategies.
- No available treatment regimen has proven to rescue the neurocognitive phenotype in humans with MCT8 deficiency. Patients require supportive/symptomatic treatment of neurological sequelae (i.e. dystonia, spasticity, drooling, scoliosis, feeding problems, epilepsy) and frequently occurring gastrointestinal symptoms (i.e. gastro-esophageal reflux disease, gastroparesis, constipation) according to common practice. In the context of MCT8 deficiency, the effectiveness of such interventions has not been evaluated.
- Being underweight in early childhood is associated with higher mortality (104), while caloric intake is frequently inadequate due to impaired swallowing function and increased catabolism due to peripheral tissue thyrotoxicosis.
- There is little literature on the management of MCT8 deficiency pre-conception or during pregnancy. A single case of prenatal, intra-amniotic treatment with high dose levothyroxine starting from gestational week 17 suggests intervention may have beneficial effects on neurodevelopmental outcomes (125).

### Areas of uncertainty and challenges

- Timely diagnoses allow early intervention with therapies (i.e. thyroid hormone analogs).
- The disorder is not diagnosed in current neonatal screening programs.
- In early life, symptoms can be non-specific.
- Awareness and knowledge of the condition among clinicians are limited.
- Cross-reactivity of TRIAC in most T3 immunoassays precludes precise measurement of serum T3 concentrations during TRIAC therapy. Alternatively, liquid chromatography with tandem mass spectrometry (LC-MS/MS) T3 assays are not susceptible to TRIAC cross-reactivity.
- Effects (and optimal dosage) of TRIAC (and other therapies) on neurocognitive outcomes, particularly when initiated early in life.
- Potential adverse effects of further lowering (F)T4 concentrations with TRIAC treatment.
- Limited access to tools that can determine the pathogenicity of (novel) variants.
- Identification of (rare) female cases with MCT8 deficiency and skewed X-inactivation.

## Selenoprotein deficiency

### Background

- Selenium, an essential trace element, is incorporated as the amino acid selenocysteine (Sec) into 25 different human selenoproteins, including the iodothyronine deiodinase enzymes. Homozygous or compound heterozygous mutations in factors required for incorporation of Sec into selenoproteins during their synthesis (Selenocysteine insertion-sequence binding protein 2, *SECISBP2*; tRNA selenocysteine (anticodon TCA) 1-1, *TRU-TCA1-1*), mediate multisystem disorders (OMIM 609698: Thyroid hormone metabolism abnormal 1; OMIM 620198: Thyroid hormone metabolism abnormal 3; ORPHA:171706) characterized by abnormal thyroid function and low plasma selenium (126, 127, 128). Diverse phenotypes are caused either by a deficiency of selenoproteins or attributable to tissue oxidative damage secondary to the loss of antioxidant selenoenzymes (129).
- Defects in another factor (O-phosphoserine-tRNA:selenocysteine tRNA synthase, SEPSECS) in this biosynthetic pathway cause a disorder with progressive microcephaly due to cortical and cerebellar atrophy, but normal circulating T4 and selenium concentrations (129).
- *SECISBP2* is a complex gene, encoding different protein isoforms, within which mutations in both coding and noncoding regions have been described; two unrelated patients with the same homozygous mutation in *TRU-TCA1-1*, have been described (129) (Supplementary Table 2).

### Diagnosis, management and treatment

- Deficiencies of selenoproteins result in a multisystem disorder, with diverse features (Table 5) attributable to the lack of tissue-specific selenoproteins, oxidative damage due to the loss of antioxidant selenoenzymes, and disordered thyroid hormone metabolism reflecting reduced activity of selenocysteine-containing deiodinases.

**Table 5:** Clinical features and investigation of selenoprotein deficiency due to *SECISBP2* mutations.

System	Clinical features	Investigation^a^
Biochemical	Abnormal thyroid function; low circulating selenoproteins	**TSH, raised free T4, normal/low free T3, highreverse T3;free T4/freeT3 ratio, high;low plasma selenium;** plasma glutathione peroxidase type 3, serum selenoprotein P
Metabolic	Increased fat mass, increased systemic insulin sensitivity	DXA scan; whole-body MRI scan; fasting glucose, insulin, and lipid profile; low hepatic lipid on MRS
Musculoskeletal	Growth retardation; axial and limb muscular dystrophy; hypoventilation	**Auxology; T1-weighted MRI** (fatty infiltration adductor, sartorius, paraspinal muscles); vital capacity; muscle biopsy (type 1 fiber predominance; disorganized sarcomeres – ‘minicores’”)
Auditory function	Hearing loss	**Audiometry;** abnormal otoacoustic emissions; normal brainstem auditory evoked responses
Cardiovascular	Thoracic aortic aneurysm	**Serial echocardiography;**MR aortogram
Reproductive	Male infertility	**Semen analysis**
Cutaneous	Photosensitivity; Raynaud’s disease	Ultraviolet A irradiation patch testing

^a^**Key investigations are in bold**
- Raised serum FT4, normal or low FT3, and raised reverse T3 concentrations, together with low plasma selenium concentrations, are biochemical hallmarks of selenoprotein deficiency due to mutations in *SECISBP2* or *TRU-TCA1-1*.
- Short stature and delayed development in childhood, whose basis is not fully understood, have been recorded in most cases (126, 130, 131, 132, 133). Weakness and hypotonia, due to progressive degeneration of specific muscle groups (e.g. sartorius, adductor, axial paraspinal) which resemble muscular dystrophy due to SELENON deficiency, is a major phenotype (129, 130, 131, 134, 135). Other phenotypes include aneurysmal dilatation of the thoracic aorta (133) sensorineural hearing loss, cutaneous photosensitivity, and male infertility (135).
- Liothyronine therapy corrects subnormal circulating FT3 concentrations (130) and improves linear growth when administered alone (130) or in combination with growth hormone (131), but untreated individuals can also attain normal height. Although oral selenium supplementation in SECISBP2 deficiency restores plasma selenium concentrations (130, 134, 136), it does not correct circulating selenoprotein deficiencies or impaired conversion of T4 to T3 (137). Antioxidant (e.g. alpha-tocopherol) treatment protects the patient’s cells and protein lipids from oxidative damage (133, 138), without adversely affecting their favorable metabolic phenotype (133, 135).

### Areas of uncertainty

- Most patients identified hitherto are children or young adults. Whether chronic oxidative tissue damage, secondary to their known reduced antioxidant defenses, predisposes to other complications (e.g. neurodegeneration, premature aging, neoplasia) at a later age remains unknown.
- Whether selenium supplementation can correct selenoprotein deficiencies in *TRU-TCA 1-1* mutation patients or whether long-term antioxidant therapies can ameliorate or prevent multisystem complications of selenoprotein deficiency remains to be determined.

## Iodothyronine deiodinase defects

### Background

- The search for mutations in the deiodinase (DIO) genes has intensified after the generation of mice deficient in each of the three deiodinases (DioKOs) (139, 140, 141, 142) (Supplementary Table 3). Until 2021, the only genetic conditions affecting deiodinases were *SECISBP2* and *TRU-TCA1-1*-dependent defects in selenoprotein synthesis (see disorders of thyroid hormone metabolism due to selenoprotein deficiency).
- Only three families with pathogenic *DIO1* mutations (OMIM 619855: Thyroid hormone metabolism abnormal 2; ORPHA:171706) have been reported (143, 144). All affected individuals were heterozygous, causing haploinsufficiency as in heterozygous *Dio1*KO mice (Supplementary Figure 3), and exhibited abnormalities including elevated circulating reverse T3, a high rT3/T3 ratio, and (in one family) raised total cholesterol concentrations.
- D1, the product of the DIO1 gene, deiodinates the outer and, to a lesser degree, the inner ring of T4, producing T3 and reverse T3 (rT3), respectively.

### Management

- Whether specific treatment or intervention is required in DIO1 variant carriers is undetermined.

### Areas of uncertainty

- The coexistence of D1 haploinsufficiency with other congenital thyroid defects may require adjustment of thyroxine replacement therapy to ensure adequate bioavailability of T3 in tissues, particularly during development.

## Supplementary Materials

Supplementary Figure 1: Compilation of known pathogenic variants in THRB. Domains of TRβ2 and TRβ1 showing that with two exceptions (light blue circles) all pathogenic TRβ variants described to date, localise to three clusters within the hormone binding domain.

Supplementary Figure 2: Compilation of known pathogenic variants in MCT8 (SLC16A2). Pathogenic variants in MCT8 (SLC16A2). Different coloured boxes depict the location of variants in transmembrane domains (TMDs) or extracellular (top) and intracellular (bottom) loops of the protein. Large deletions (lines) and splice site variants (arrowheads) are superimposed on the genomic organisation or SLC16A2 (bottom of picture).

Supplementary Figure 3: Phenotype of known DIO1 mutations. Serum rT3 concentrations and rT3/T3 ratios in affected (closed circles) and unaffected (open circles) members of two families with DIO1 mutations. Deiodinase enzyme activity, measured in cells expressing either the WT and two D1 mutants alone or in combination with WT (simulating the heterozygous status of patients), is shown.

Supplementary Table 1: Compilation of all published pathogenic variants in THRA

Supplementary Table 2: Compilation of all published pathogenic variants in SECISBP2 and TRU-TCA1-1

Supplementary Table 3: Phenotype of mice deficient in deiodinases

## Declaration of interest

The task force had no commercial support and LP, PR, SR, MG, PBP, and KC have no conflicts of interest to declare. CM has consulted for Egetis Therapeutics. The Erasmus Medical Centre (Rotterdam, Netherlands), which employs RP, WEV, and SG, receives royalties from Egetis Therapeutics (a manufacturer of TRIAC), dependent on commercialization. None of the authors will benefit personally from any royalties.

## Funding

MG and KC are supported by the NIHR Cambridge Biomedical Research Centrehttp://dx.doi.org/10.13039/501100018956 (NIHR203312). KC is supported by a Wellcome Trusthttp://dx.doi.org/10.13039/100010269 Investigator Award (210755/Z/18/Z) and Medical Research Councilhttp://dx.doi.org/10.13039/501100000265 funding (MRC_MC_UU_00014/40). SR is supported by a grant (DK15070) from the National Institutes of Healthhttp://dx.doi.org/10.13039/100000002, USA. LP is supported by funding from the Italian Ministry of Healthhttp://dx.doi.org/10.13039/100009647, Rome, Italy (Ricerca Corrente and PNRR-MR1-2022-12375726; RTH2018, 05C821_2018). PR is supported by the plan maladies rares of the French health care ministry. WEV is supported by ZonMW (Dutch Research Council, VIDI 09150172210071). LP, PR, WEV, SG, and RP are members of the European Reference Network on Rare Endocrine Conditions (Endo-ERN), which is co-funded by the European Union’s 3rd Health Programme (CHAFEA Framework Partnership agreement no. 739527).

## References

[1] Refetoff S Bassett JHD Beck-Peccoz P Bernal J Brent G Chatterjee K De Groot LJ Dumitrescu AM Jameson JL Kopp PA, *et al.* Classification and proposed nomenclature for inherited defects of thyroid hormone action, cell transport, and metabolism. Thyroid 2014 24 407–409. (10.1089/thy.2013.3393.nomen) 24588711 PMC3950730

[2] Refetoff S Bassett JHD Beck-Peccoz P Bernal J Brent G Chatterjee K De Groot LJ Dumitrescu AM Jameson JL Kopp PA, *et al.* Classification and proposed nomenclature for inherited defects of thyroid hormone action, cell transport, and metabolism. Journal of Clinical Endocrinology and Metabolism 2014 99 768–770. (10.1210/jc.2013-3393) 24823702 PMC3942236

[3] Refetoff S Bassett JH Beck-Peccoz P Bernal J Brent G Chatterjee K De Groot LJ Dumitrescu AM Jameson JL Kopp PA, *et al.* Classification and proposed nomenclature for inherited defects of thyroid hormone action, cell transport, and metabolism. Thyroid 2014 24 407–409. (10.1089/thy.2013.3393.nomen) 24588711 PMC3950730

[4] Atkins D Eccles M Flottorp S Guyatt GH Henry D Hill S Liberati A O'Connell D Oxman AD Phillips Bet al. Systems for grading the quality of evidence and the strength of recommendations I: critical appraisal of existing approaches The GRADE Working Group. *BMC Health Services Research* 2004 4 38. (10.1186/1472-6963-4-38) PMC545647 15615589

[5] Swiglo BA Murad MH Schünemann HJ Kunz R Vigersky RA Guyatt GH & Montori VM. A case for clarity, consistency, and helpfulness: state-of-the-art clinical practice guidelines in endocrinology using the grading of recommendations, assessment, develoment, and evaluation system. Journal of Clinical Endocrinology and Metabolism 2008 93 666–673. (10.1210/jc.2007-1907) 18171699

[6] Koulouri O Moran C Halsall D Chatterjee K & Gurnell M. Pitfalls in the measurement and interpretation of thyroid function tests. Best Practice and Research. Clinical Endocrinology and Metabolism 2013 27 745–762. (10.1016/j.beem.2013.10.003) 24275187 PMC3857600

[7] Moran C Schoenmakers N Halsall D Oddy S Lyons G van den Berg S Gurnell M & Chatterjee K. Approach to the patient with raised thyroid hormones and non-suppressed TSH. Journal of Clinical Endocrinology and Metabolism 2024 109 1094–1108. (10.1210/clinem/dgad681) 37988295 PMC10940260

[8] Favresse J Burlacu MC Maiter D & Gruson D. Interferences with thyroid function immunoassays: clinical implications and detection algorithm. Endocrine Reviews 2018 39 830–850. (10.1210/er.2018-00119) 29982406

[9] Beck-Peccoz P Lania A Beckers A Chatterjee K & Wemeau JL. 2013 European Thyroid Association guidelines for the diagnosis and treatment of thyrotropin-secreting pituitary tumors. European Thyroid Journal 2013 2 76–82. (10.1159/000351007) 24783044 PMC3821512

[10] Gurnell M Bashari WA Senanayake R MacFarlane J & Koulouri O Thyroid Stimulating Hormone Producing Pituitary Tumours. In: De Groot’s Endocrinology, 8th ed. Eds. Robertson RP Giudice LC Grossman AB, *et al.*, pp. 145–154. Elsevier 2022.

[11] Dumitrescu AM & Refetoff S. The syndromes of reduced sensitivity to thyroid hormone. Biochimica et Biophysica Acta 2013 1830 3987–4003. (10.1016/j.bbagen.2012.08.005) 22986150 PMC3528849

[12] Moran C Schoenmakers N Visser WE Schoenmakers E Agostini M & Chatterjee K. Genetic disorders of thyroid development, hormone biosynthesis and signalling. Clinical Endocrinology 2022 97 502–514. (10.1111/cen.14817) 35999191 PMC9544560

[13] Persani L & Campi I. Syndromes of resistance to thyroid hormone action. Experientia Supplementum 2019 111 55–84. (10.1007/978-3-030-25905-1_5) 31588528

[14] Refetoff S Weiss RE & Usala SJ. The syndromes of resistance to thyroid hormone. Endocrine Reviews 1993 14 348–399. (10.1210/edrv-14-3-348) 8319599

[15] Mamanasiri S Yesil S Dumitrescu AM Liao XH Demir T Weiss RE & Refetoff S. Mosaicism of a thyroid hormone receptor-beta gene mutation in resistance to thyroid hormone. Journal of Clinical Endocrinology and Metabolism 2006 91 3471–3477. (10.1210/jc.2006-0727) 16804041

[16] Donnars A Leplat A Grosheny C Briet C Illouz F Bouzamondo N Moal V De Casson FB Bouhours-Nouet N Coutant R, *et al.* Clinically symptomatic resistance to thyroid hormone beta syndrome because of THRB gene mosaicism. Journal of Clinical Endocrinology and Metabolism 2022 107 e3548–e3552. (10.1210/clinem/dgac347) 35689814

[17] Chatterjee VK Nagaya T Madison LD Datta S Rentoumis A & Jameson JL. Thyroid hormone resistance syndrome. Inhibition of normal receptor function by mutant thyroid hormone receptors. Journal of Clinical Investigation 1991 87 1977–1984. (10.1172/JCI115225) 2040690 PMC296951

[18] Adams M Matthews C Collingwood TN Tone Y Beck-Peccoz P & Chatterjee KK. Genetic analysis of 29 kindreds with generalized and pituitary resistance to thyroid hormone. Identification of thirteen novel mutations in the thyroid hormone receptor beta gene. Journal of Clinical Investigation 1994 94 506–515. (10.1172/JCI117362) 8040303 PMC296123

[19] Collingwood TN Adams M Tone Y & Chatterjee VK. Spectrum of transcriptional, dimerization, and dominant negative properties of twenty different mutant thyroid hormone beta-receptors in thyroid hormone resistance syndrome. Molecular Endocrinology 1994 8 1262–1277. (10.1210/mend.8.9.7838159) 7838159

[20] Yoh SM Chatterjee VK & Privalsky ML. Thyroid hormone resistance syndrome manifests as an aberrant interaction between mutant T3 receptors and transcriptional corepressors. Molecular Endocrinology 1997 11 470–480. (10.1210/mend.11.4.9914) 9092799 PMC2725002

[21] Collingwood TN Rajanayagam O Adams M Wagner R Cavailles V Kalkhoven E Matthews C Nystrom E Stenlof K Lindstedt G, *et al.* A natural transactivation mutation in the thyroid hormone beta receptor: impaired interaction with putative transcriptional mediators. PNAS 1997 94 248–253. (10.1073/pnas.94.1.248) 8990194 PMC19304

[22] Wu SY Cohen RN Simsek E Senses DA Yar NE Grasberger H Noel J Refetoff S & Weiss RE. A novel thyroid hormone receptor-beta mutation that fails to bind nuclear receptor corepressor in a patient as an apparent cause of severe, predominantly pituitary resistance to thyroid hormone. Journal of Clinical Endocrinology and Metabolism 2006 91 1887–1895. (10.1210/jc.2005-2428) 16464943

[23] Wejaphikul K Groeneweg S Dejkhamron P Unachak K Visser WE Chatterjee VK Visser TJ Meima ME & Peeters RP. Role of leucine 341 in thyroid hormone receptor beta revealed by a novel mutation causing thyroid hormone resistance. Thyroid 2018 28 1723–1726. (10.1089/thy.2018.0146) 30362879

[24] Abdellaoui Y Magkou D Bakopoulou S Zaharia R Raffin-Sanson ML & Cazabat L. Coexistence of autoimmune hyper- and hypothyroidism in a kindred with reduced sensitivity to thyroid hormone. European Thyroid Journal 2020 9 263–268. (10.1159/000506424) 33088795 PMC7548835

[25] Campi I Covelli D Moran C Fugazzola L Cacciatore C Orlandi F Gallone G Chatterjee K Beck-Peccoz P & Persani L. The differential diagnosis of discrepant thyroid function tests: insistent pitfalls and updated flow-chart based on a long-standing experience. Frontiers in Endocrinology (Lausanne) 2020 11 432. (10.3389/fendo.2020.00432) PMC7358450 32733382

[26] Larsen CC Dumitrescu A Guerra-Arguero LM Gallego-Suarez C Vazquez-Mellado A Vinogradova M Fletterick R Refetoff S & Weiss RE. Incidental identification of a thyroid hormone receptor beta (THRB) gene variant in a family with autoimmune thyroid disease. Thyroid 2013 23 1638–1643. (10.1089/thy.2013.0174) 23806029 PMC3868256

[27] Okazaki-Hada M Nishihara E Hisakado M Kudo T Ito M Fukata S Nishikawa M Akamizu T & Miyauchi A. Autoimmune thyroid disease and thyroid function test fluctuations in patients with resistance to thyroid hormone. European Journal of Endocrinology 2021 186 73–82. (10.1530/EJE-21-0584) 34727089

[28] Borck G Seewi O Jung A Schonau E & Kubisch C. Genetic causes of goiter and deafness: Pendred syndrome in a girl and cooccurrence of Pendred syndrome and resistance to thyroid hormone in her sister. Journal of Clinical Endocrinology and Metabolism 2009 94 2106–2109. (10.1210/jc.2008-2361) 19318451

[29] Grasberger H Ringkananont U Croxson M & Refetoff S. Resistance to thyroid hormone in a patient with thyroid dysgenesis. Thyroid 2005 15 730–733. (10.1089/thy.2005.15.730) 16053391

[30] Lauffer P Bikker H Garrelfs MR Hillebrand JJG de Sonnaville MCS Zwaveling-Soonawala N & van Trotsenburg ASP. Defective levothyroxine response in a patient with dyshormonogenic congenital hypothyroidism caused by a concurrent pathogenic variant in thyroid hormone receptor-beta. Thyroid 2021 31 1757–1762. (10.1089/thy.2021.0204) 34382419

[31] Salas-Lucia F Franca MM Amrhein JA Weir JE Dumitrescu AM & Refetoff S. Severe resistance to thyroid hormone beta in a patient with athyreosis. Thyroid 2022 32 336–339. (10.1089/thy.2021.0523) 34969265 PMC8971974

[32] Sato H. Clinical features of primary hyperthyroidism caused by Graves' disease admixed with resistance to thyroid hormone (P453T). Endocrine Journal 2010 57 687–692. (10.1507/endocrj.k10e-066) 20574139

[33] Sivakumar T & Chaidarun S. Resistance to thyroid hormone in a patient with coexisting Graves' disease. Thyroid 2010 20 213–216. (10.1089/thy.2009.0175) 20151830

[34] Brucker-Davis F Skarulis MC Grace MB Benichou J Hauser P Wiggs E & Weintraub BD. Genetic and clinical features of 42 kindreds with resistance to thyroid hormone. The National Institutes of Health prospective study. Annals of Internal Medicine 1995 123 572–583. (10.7326/0003-4819-123-8-199510150-00002) 7677297

[35] Persani L Asteria C Tonacchera M Vitti P Krishna V Chatterjee K & Beck-Peccoz P. Evidence for the secretion of thyrotropin with enhanced bioactivity in syndromes of thyroid hormone resistance. Journal of Clinical Endocrinology and Metabolism 1994 78 1034–1039. (10.1210/jcem.78.5.8175956) 8175956

[36] Igata M Tsuruzoe K Kawashima J Kukidome D Kondo T Motoshima H Shimoda S Furukawa N Nishikawa T Miyamura N, *et al.* Coexistence of resistance to thyroid hormone and papillary thyroid carcinoma. Endocrinology, Diabetes and Metabolism Case Reports 2016 2016 160003. (10.1530/EDM-16-0003) PMC4861829 27168936

[37] Ramos-Prol A Antonia Perez-Lazaro M Isabel del Olmo-Garcia M Leon-de Zayas B Moreno-Macian F Navas-de Solis S & Merino-Torres JF. Differentiated thyroid carcinoma in a girl with resistance to thyroid hormone management with triiodothyroacetic acid. Journal of Pediatric Endocrinology and Metabolism 2013 26 133–136. (10.1515/jpem-2012-0230) 23457315

[38] Unluturk U Sriphrapradang C Erdogan MF Emral R Guldiken S Refetoff S & Gullu S. Management of differentiated thyroid cancer in the presence of resistance to thyroid hormone and TSH-secreting adenomas: a report of four cases and review of the literature. Journal of Clinical Endocrinology and Metabolism 2013 98 2210–2217. (10.1210/jc.2012-4142) 23553855 PMC3667261

[39] Vinagre J Borges F Costa A Alvelos MI Mazeto G Sobrinho-Simoes M & Soares P. Differentiated thyroid cancer in patients with resistance to thyroid hormone syndrome. A novel case and a review of the literature. Frontiers in Molecular Biosciences 2014 **1** 10 1. (10.3389/fmolb.2014.00010) PMC4429638 25988151

[40] Barkoff MS Kocherginsky M Anselmo J Weiss RE & Refetoff S. Autoimmunity in patients with resistance to thyroid hormone. Journal of Clinical Endocrinology and Metabolism 2010 95 3189–3193. (10.1210/jc.2009-2179) 20444926 PMC2928894

[41] Illouz F Briet C Mirebeau-Prunier D Bouhours-Nouet N Coutant R Sibilia P & Rodien P. Cardiac complications of thyroid hormone resistance syndromes. Annales d’Endocrinologie 2021 82 167–169. (10.1016/j.ando.2020.03.008) 32513415

[42] Kahaly GJ Matthews CH Mohr-Kahaly S Richards CA & Chatterjee VKK. Cardiac involvement in thyroid hormone resistance. Journal of Clinical Endocrinology and Metabolism 2002 87 204–212. (10.1210/jcem.87.1.8170) 11788648

[43] Kurozumi A Okada Y Arao T & Tanaka Y. A case of resistance to thyroid hormone (RTH) with a negative family history with diagnosis based on persistent palpitations. Journal of UOEH 2016 38 291–296. (10.7888/juoeh.38.291) 27980311

[44] Pulcrano M Palmieri EA Mannavola D Ciulla M Campi I Covelli D Lombardi G Biondi B & Beck-Peccoz P. Impact of resistance to thyroid hormone on the cardiovascular system in adults. Journal of Clinical Endocrinology and Metabolism 2009 94 2812–2816. (10.1210/jc.2009-0096) 19435825

[45] Okosieme OE Usman D Taylor PN Dayan CM Lyons G Moran C Chatterjee K & Rees DA. Cardiovascular morbidity and mortality in patients in Wales, UK with resistance to thyroid hormone beta (RTHbeta): a linked-record cohort study. Lancet Diabetes and Endocrinology 2023 11 657–666. 37475119 10.1016/S2213-8587(23)00155-9

[46] Beck-Peccoz P Roncoroni R Mariotti S Medri G Marcocci C Brabant G Forloni F Pinchera A & Faglia G. Sex hormone-binding globulin measurement in patients with inappropriate secretion of thyrotropin (IST): evidence against selective pituitary thyroid hormone resistance in nonneoplastic IST. Journal of Clinical Endocrinology and Metabolism 1990 71 19–25. (10.1210/jcem-71-1-19) 2370293

[47] Sarne DH Refetoff S Rosenfield RL & Farriaux JP. Sex hormone-binding globulin in the diagnosis of peripheral tissue resistance to thyroid hormone: the value of changes after short term triiodothyronine administration. Journal of Clinical Endocrinology and Metabolism 1988 66 740–746. (10.1210/jcem-66-4-740) 3346353

[48] Chaves C Bruinstroop E Refetoff S Yen PM & Anselmo J. Increased hepatic fat content in patients with resistance to thyroid hormone beta. Thyroid 2021 31 1127–1134. (10.1089/thy.2020.0651) 33353459 PMC8290309

[49] Laclaustra M Moreno-Franco B Lou-Bonafonte JM Mateo-Gallego R Casasnovas JA Guallar-Castillon P Cenarro A & Civeira F. Impaired sensitivity to thyroid hormones is associated with diabetes and metabolic syndrome. Diabetes Care 2019 42 303–310. (10.2337/dc18-1410) 30552134

[50] Moran C McEniery CM Schoenmakers N Mitchell C Sleigh A Watson L Lyons G Burling K Barker P & Chatterjee K. Dyslipidemia, insulin Resistance, ectopic Lipid Accumulation, and Vascular Function in Resistance to thyroid Hormone beta. Journal of Clinical Endocrinology and Metabolism 2021 106 e2005–e2014. (10.1210/clinem/dgab002) 33524107 PMC8063262

[51] Wakasaki H Matsumoto M Tamaki S Miyata K Yamamoto S Minaga T Hayashi Y Komukai K Imanishi T Yamaoka H, *et al.* Resistance to thyroid hormone complicated with type 2 diabetes and cardiomyopathy in a patient with a TRbeta mutation. Internal Medicine 2016 55 3295–3299. (10.2169/internalmedicine.55.7147) 27853072 PMC5173497

[52] Hauser P Zametkin AJ Martinez P Vitiello B Matochik JA Mixson AJ & Weintraub BD. Attention deficit-hyperactivity disorder in people with generalized resistance to thyroid hormone. New England Journal of Medicine 1993 328 997–1001. (10.1056/NEJM199304083281403) 8450877

[53] Uter J Heldmann M Rogge B Obst M Steinhardt J Brabant G Moran C Chatterjee K & Munte TF. Patients with mutations of the thyroid hormone beta-receptor show an ADHD-like phenotype for performance monitoring: an electrophysiological study. NeuroImage. Clinical 2020 26 102250. (10.1016/j.nicl.2020.102250) 32217468 PMC7109456

[54] Mixson AJ Parrilla R Ransom SC Wiggs EA McClaskey JH Hauser P & Weintraub BD. Correlations of language abnormalities with localization of mutations in the beta-thyroid hormone receptor in 13 kindreds with generalized resistance to thyroid hormone: identification of four new mutations. Journal of Clinical Endocrinology and Metabolism 1992 75 1039–1045. (10.1210/jcem.75.4.1400869) 1400869

[55] Stein MA Weiss RE & Refetoff S. Neurocognitive characteristics of individuals with resistance to thyroid hormone: comparisons with individuals with attention-deficit hyperactivity disorder. Journal of Developmental and Behavioral Pediatrics 1995 16 406–411. (10.1097/00004703-199512000-00003) 8746549

[56] Ferrara AM Onigata K Ercan O Woodhead H Weiss RE & Refetoff S. Homozygous thyroid hormone receptor beta-gene mutations in resistance to thyroid hormone: three new cases and review of the literature. Journal of Clinical Endocrinology and Metabolism 2012 97 1328–1336. (10.1210/jc.2011-2642) 22319036 PMC3319181

[57] Brucker-Davis F Skarulis MC Pikus A Ishizawar D Mastroianni MA Koby M & Weintraub BD. Prevalence and mechanisms of hearing loss in patients with resistance to thyroid hormone. Journal of Clinical Endocrinology and Metabolism 1996 81 2768–2772. (10.1210/jcem.81.8.8768826) 8768826

[58] Campi I Cammarata G Bianchi Marzoli S Beck-Peccoz P Santarsiero D Dazzi D Bottari de Castello A Taroni EG Viola F Mian C, *et al.* Retinal photoreceptor functions are compromised in patients with resistance to thyroid hormone syndrome (RTHbeta). Journal of Clinical Endocrinology and Metabolism 2017 102 2620–2627. (10.1210/jc.2016-3671) 28379567

[59] Refetoff S DeWind LT & DeGroot LJ. Familial syndrome combining deaf-mutism, stuppled epiphyses, goiter and abnormally high PBI: possible target organ refractoriness to thyroid hormone. Journal of Clinical Endocrinology and Metabolism 1967 27 279–294. (10.1210/jcem-27-2-279) 4163616

[60] Weiss AH Kelly JP Bisset D & Deeb SS. Reduced L- and M- and increased S-cone functions in an infant with thyroid hormone resistance due to mutations in the THRbeta2 gene. Ophthalmic Genetics 2012 33 187–195. (10.3109/13816810.2012.681096) 22551329

[61] Fernández-Suárez E González-Del Pozo M García-Núñez A Méndez-Vidal C Martín-Sánchez M Mejías-Carrasco JM Ramos-Jiménez M Morillo-Sánchez MJ Rodríguez-de la Rúa E Borrego S , *et al.* Expanding the phenotype of *THRB*: a range of macular dystrophies as the major clinical manifestations in patients with a dominant splicing variant. Frontiers in Cell and Developmental Biology 2023 11 1197744. (10.3389/fcell.2023.1197744) 37547476 PMC10401274

[62] Anselmo J Cao D Karrison T Weiss RE & Refetoff S. Fetal loss associated with excess thyroid hormone exposure. JAMA 2004 292 691–695. (10.1001/jama.292.6.691) 15304465

[63] Pappa T Anselmo J Mamanasiri S Dumitrescu AM Weiss RE & Refetoff S. Prenatal diagnosis of resistance to thyroid hormone and its clinical implications. Journal of Clinical Endocrinology and Metabolism 2017 102 3775–3782. (10.1210/jc.2017-01251) 28938413 PMC5630247

[64] Blair JC Mohan U Larcher VF Rajanayagam O Burrin JM Perry LA Grossman AB Chatterjee VK & Savage MO. Neonatal thyrotoxicosis and maternal infertility in thyroid hormone resistance due to a mutation in the TRbeta gene (M313T). Clinical Endocrinology 2002 57 405–409. (10.1046/j.1365-2265.2002.01588.x) 12201835

[65] Weiss RE Dumitrescu A & Refetoff S. Approach to the patient with resistance to thyroid hormone and pregnancy. Journal of Clinical Endocrinology and Metabolism 2010 95 3094–3102. (10.1210/jc.2010-0409) 20610605 PMC2928892

[66] Beck-Peccoz P & Chatterjee VK. The variable clinical phenotype in thyroid hormone resistance syndrome. Thyroid 1994 4 225–232. (10.1089/thy.1994.4.225) 7920008

[67] Hayashi Y Weiss RE Sarne DH Yen PM Sunthornthepvarakul T Marcocci C Chin WW & Refetoff S. Do clinical manifestations of resistance to thyroid hormone correlate with the functional alteration of the corresponding mutant thyroid hormone-beta receptors? Journal of Clinical Endocrinology and Metabolism 1995 80 3246–3256. (10.1210/jcem.80.11.7593433) 7593433

[68] Mitchell CS Savage DB Dufour S Schoenmakers N Murgatroyd P Befroy D Halsall D Northcott S Raymond-Barker P Curran S , *et al.* Resistance to thyroid hormone is associated with raised energy expenditure, muscle mitochondrial uncoupling, and hyperphagia. Journal of Clinical Investigation 2010 120 1345–1354. (10.1172/JCI38793) 20237409 PMC2846038

[69] Usala SJ Menke JB Watson TL Wondisford FE Weintraub BD Berard J Bradley WE Ono S Mueller OT & Bercu BB. A homozygous deletion in the c-erbA beta thyroid hormone receptor gene in a patient with generalized thyroid hormone resistance: isolation and characterization of the mutant receptor. Molecular Endocrinology 1991 5 327–335. (10.1210/mend-5-3-327) 1653889

[70] Moran C Habeb AM Kahaly GJ Kampmann C Hughes M Marek J Rajanayagam O Kuczynski A Vargha-Khadem F Morsy M, *et al.* Homozygous resistance to thyroid hormone beta: can combined antithyroid drug and triiodothyroacetic acid treatment prevent cardiac failure? Journal of the Endocrine Society 2017 1 1203–1212. (10.1210/js.2017-00204) 29264576 PMC5686666

[71] Aguilar Diosdado M Escobar-Jimenez L Fernandez Soto ML Garcia Curiel A & Escobar-Jimenez F. Hyperthyroidism due to familial pituitary resistance to thyroid hormone: successful control with 3, 5, 3' triiodothyroacetic associated to propranolol. Journal of Endocrinological Investigation 1991 14 663–668. (10.1007/BF03347890) 1774450

[72] Radetti G Persani L Molinaro G Mannavola D Cortelazzi D Chatterjee VK & Beck-Peccoz P. Clinical and hormonal outcome after two years of triiodothyroacetic acid treatment in a child with thyroid hormone resistance. Thyroid 1997 7 775–778. (10.1089/thy.1997.7.775) 9349583

[73] Takeda T Suzuki S Liu RT & DeGroot LJ. Triiodothyroacetic acid has unique potential for therapy of resistance to thyroid hormone. Journal of Clinical Endocrinology and Metabolism 1995 80 2033–2040. (10.1210/jcem.80.7.7608251) 7608251

[74] Ueda S Takamatsu J Fukata S Tanaka K Shimizu N Sakata S Yamaji T Kuma K & Ohsawa N. Differences in response of thyrotropin to 3,5,3'-triiodothyronine and 3,5,3'-triiodothyroacetic acid in patients with resistance to thyroid hormone. Thyroid 1996 6 563–570. (10.1089/thy.1996.6.563) 9001190

[75] Groeneweg S Peeters RP Visser TJ & Visser WE. Therapeutic applications of thyroid hormone analogues in resistance to thyroid hormone (RTH) syndromes. Molecular and Cellular Endocrinology 2017 458 82–90. (10.1016/j.mce.2017.02.029) 28235578

[76] Anzai R Adachi M Sho N Muroya K Asakura Y & Onigata K. Long-term 3,5,3'-triiodothyroacetic acid therapy in a child with hyperthyroidism caused by thyroid hormone resistance: pharmacological study and therapeutic recommendations. Thyroid 2012 22 1069–1075. (10.1089/thy.2011.0450) 22947347

[77] Torre P Bertoli M Di Giovanni S Scommegna S Conte C Novelli G & Cianfarani S. Endocrine and neuropsychological assessment in a child with a novel mutation of thyroid hormone receptor: response to 12-month triiodothyroacetic acid (TRIAC) therapy. Journal of Endocrinological Investigation 2005 28 657–662. (10.1007/BF03347267) 16218051

[78] Anselmo J & Refetoff S. Regression of a large goiter in a patient with resistance to thyroid hormone by every other day treatment with triiodothyronine. Thyroid 2004 14 71–74. (10.1089/105072504322783876) 15009917

[79] Weiss RE Stein MA & Refetoff S. Behavioural effects of liothyronine (L-T3) in children with attention-deficit hyperactivity disorder in the presence and absence of Resistance to thyroid Hormone. Thyroid 1997 7 389–393. (10.1089/thy.1997.7.389) 9226208

[80] Kannan S & Safer JD. Finding the right balance between resistance and sensitivity: a review of the cardiac manifestations of the syndrome of resistance to thyroid hormone and the implications for treatment. Endocrine Practice 2012 18 252–255. (10.4158/EP11075.RA) 22068246

[81] Gurnell M Rajanayagam O Barbar I Keston-Jones MK & Chatterjee VKK. Reversible pituitary enlargement in the syndrome of resistance to thyroid hormone. Thyroid 1998 8 679–682. (10.1089/thy.1998.8.679) 9737363

[82] Bochukova E Schoenmakers N Agostini M Schoenmakers E Rajanayagam O Keogh JM Henning E Reinemund J Gevers E Sarri M, *et al.* A mutation in the thyroid hormone receptor alpha gene. New England Journal of Medicine 2012 366 243–249. (10.1056/NEJMoa1110296) 22168587

[83] van Mullem A van Heerebeek R Chrysis D Visser E Medici M Andrikoula M Tsatsoulis A Peeters R & Visser TJ. Clinical phenotype and mutant TRα1. New England Journal of Medicine 2012 366 1451–1453. (10.1056/NEJMc1113940) 22494134

[84] van Mullem AA Chrysis D Eythimiadou A Chroni E Tsatsoulis A de Rijke YB Visser WE Visser TJ & Peeters RP. Clinical phenotype of a new type of thyroid hormone resistance caused by a mutation of the TRα1 receptor: consequences of LT4 treatment. Journal of Clinical Endocrinology and Metabolism 2013 98 3029–3038. (10.1210/jc.2013-1050) 23633213

[85] Moran C Schoenmakers N Agostini M Schoenmakers E Offiah A Kydd A Kahaly G Mohr-Kahaly S Rajanayagam O Lyons G, *et al.* An adult female with resistance to thyroid hormone mediated by defective thyroid hormone receptor α. Journal of Clinical Endocrinology and Metabolism 2013 98 4254–4261. (10.1210/jc.2013-2215) 23940126

[86] Moran C Agostini M Visser E Schoenmakers E Schoenmakers N Offiah AC Poole K Rajanayagam O Lyons G Halsall D, *et al.* Resistance to thyroid hormone caused by a mutation in thyroid hormone receptor (TR) alpha1 and alpha2: clinical, biochemical and genetic analyses of three related patients. Lancet Diabetes and Endocrinology 2014 2 619–626. 24969835 10.1016/S2213-8587(14)70111-1 PMC5989926

[87] Tylki-Szymańska A Acuna-Hidalgo R Krajewska-Walasek M Lecka-Ambroziak A Steehouwer M Gilissen C Brunner HG Jurecka A Różdżyńska-Świątkowska A Hoischen A , *et al.* Thyroid hormone resistance syndrome due to mutations in the thyroid hormone receptor α gene (THRA). Journal of Medical Genetics 2015 52 312–316. (10.1136/jmedgenet-2014-102936) 25670821

[88] Yuen RKC Thiruvahindrapuram B Merico D Walker S Tammimies K Hoang N Chrysler C Nalpathamkalam T Pellecchia G Liu Y, *et al.* Whole genome sequencing of Quartet families with autism spectrum disorder. Nature Medicine 2015 21 185–191. (10.1038/nm.3792) 25621899

[89] Espiard S Savagner F Flamant F Vlaeminck-Guillem V Guyot R Munier M d'Herbomez M Bourguet W Pinto G Rose C, *et al.* A novel mutation in THRA gene associated with an atypical phenotype of resistance to thyroid hormone. Journal of Clinical Endocrinology and Metabolism 2015 100 2841–2848. (10.1210/jc.2015-1120) 26037512

[90] Van Gucht ALM Meima ME Zwaveling-Soonawala N Visser WE Fliers E Wennink JMB Henny C Visser TJ Peeters RP & van Trotsenburg ASP. Resistance to thyroid hormone alpha in an 18-month-old girl: clinical, therapeutic and molecular characteristics. Thyroid 2016 26 338–346. (10.1089/thy.2015.0463) 26782358

[91] Demir K van Gucht ALM Buyukinan M Catli G Ahan Y Bas VN Dundar B Ozkan B Meima ME Visser WE, *et al.* Diverse genotypes and phenotypes of three novel thyroid hormone receptor alpha mutations. Journal of Clinical Endocrinology and Metabolism 2016 101 2945–2954. (10.1210/jc.2016-1404) 27144938

[92] Van Gucht ALM Moran C Meima ME Visser WE Chatterjee K Visser TJ & Peeters RP. Resistance to thyroid hormone due to heterozygous mutation in thyroid hormone receptor alpha. Current Topics in Developmental Biology 2017 125 337–355. (10.1016/bs.ctdb.2017.02.001) 28527577

[93] Moran C Agostini M McGowan A Schoenmakers E Fairall L Lyons G Rajanayagam O Watson L Offiah A Barton J, *et al.* Contrasting phenotype in resistance to thyroid hormone alpha correlate with divergent properties of thyroid hormone receptor a1 mutant proteins. Thyroid 2017 27 973–982. (10.1089/thy.2017.0157) 28471274 PMC5561448

[94] Wejaphikul K Groeneweg S Hilhorst-Hofsee Y Chatterjee VK Peeters RP Meima ME & Visser WE. Insight into molecular determinants of T3 versus T4 recognition from mutations in thyroid hormone receptor alpha and beta. Journal of Clinical Endocrinology and Metabolism 2019 104 3491–3500. (10.1210/jc.2018-02794) 30817817 PMC6599431

[95] Korkmaz O Ozen S Ozdemir TR Goksen D & Darcan S. A novel thyroid hormone receptor alpha gene mutation, clinic characteristics, and follow-up findings in a patient with thyroid hormone resistance. Hormones 2019 18 223–227. (10.1007/s42000-019-00094-9) 30747412

[96] Sun H Wu H Xie R Wang F Chen T Chen X Wang X Flamant F & Chen L. New case of thyroid hormone resistance a caused by a mutation of THRA/Tra1. Journal of the Endocrine Society 2019 3 665–669. 30842990 10.1210/js.2019-00011 PMC6397419

[97] Le Maire A Bouhours-Nouet N Soamalala J Mirebeau-Prunier D Paloni M Guee L Heron D Mignot C Illouz F Joubert F, *et al.* Two novel cases of resistance to thyroid hormone due to THRA mutation. Thyroid 2020 30 1217–1221. (10.1089/thy.2019.0602) 32204686

[98] Furman AE Dumitrescu AM Refetoff S & Weiss RE. Early diagnosis and treatment of an infant with a novel thyroid hormone receptor alpha gene (cC380SfsX9) mutation. Thyroid 2021 31 1003–1005. (10.1089/thy.2020.0695) 33198587 PMC8215396

[99] Al Shidhani A Ullah I AlSaffar H Kindi AA Al Nabhani H & Al Yaarubi S. Thyroid hormone resistance due to a novel de novo mutation in thyroid hormone receptor alpha: first case report from the Middle East and North Africa. Oman Medical Journal 2021 36 e226. (10.5001/omj.2021.20) 33628462 PMC7897355

[100] Dahll LK Westbye AB Vinorum K Sejersted Y Barøy T Thorsby PM & Hammerstad SS. Clinical and Biochemical characteristics of untreated adult patients with resistance to thyroid hormone alpha. Journal of the Endocrine Society 2023 7 bvad089. (10.1210/jendso/bvad089) 37469961 PMC10353041

[101] Erbas IM Çakir MD Yener AS & Demir K. Long-term follow-up results and treatment outcomes of children and adults with resistance to thyroid hormone alpha. Journal of Endocrinological Investigation 2023 46 1855–1863. (10.1007/s40618-023-02043-1) 36821077

[102] Dore R Watson L Hollidge S Krause C Sentis SC Oelkrug R Geißler C Johann K Pedaran M Lyons G, *et al.* Resistance to thyroid hormone induced tachycardia in RTH alpha syndrome. Nature Communications 2023 14 3312. (10.1038/s41467-023-38960-1) PMC10247713 37286550

[103] Moran C & Chatterjee K. Resistance to thyroid hormone due to defective thyroid receptor alpha. Best Practice and Research. Clinical Endocrinology and Metabolism 2015 29 647–657. (10.1016/j.beem.2015.07.007) 26303090 PMC4559105

[104] Groeneweg S van Geest FS Abaci A Alcantud A Ambegaonkar GP Armour CM Bakhtiani P Barca D Bertini ES van Beynum IM, *et al.* Disease characteristics of MCT8 deficiency: an international, retrospective, multicentre cohort study. Lancet. Diabetes and Endocrinology 2020 8 594–605. (10.1016/S2213-8587(2030153-4) 32559475 PMC7611932

[105] Biebermann H Ambrugger P Tarnow P von Moers A Schweizer U & Grueters A. Extended clinical phenotype, endocrine investigations and functional studies of a loss-of-function mutation A150V in the thyroid hormone specific transporter MCT8. European Journal of Endocrinology 2005 153 359–366. (10.1530/eje.1.01980) 16131597

[106] Kim JH Kim YM Yum MS Choi JH Lee BH Kim GH & Yoo HW. Clinical and endocrine features of two Allan-Herndon-Dudley syndrome patients with monocarboxylate transporter 8 mutations. Hormone Research in Paediatrics 2015 83 288–292. (10.1159/000371466) 25896225

[107] Shimojima K Maruyama K Kikuchi M Imai A Inoue K & Yamamoto T. Novel SLC16A2 mutations in patients with Allan-Herndon-Dudley syndrome. Intractable and Rare Diseases Research 2016 5 214–217. (10.5582/irdr.2016.01051) 27672545 PMC4995413

[108] Fuchs O Pfarr N Pohlenz J & Schmidt H. Elevated serum triiodothyronine and intellectual and motor disability with paroxysmal dyskinesia caused by a monocarboxylate transporter 8 gene mutation. Developmental Medicine and Child Neurology 2009 51 240–244. (10.1111/j.1469-8749.2008.03125.x) 19018842

[109] Novara F Groeneweg S Freri E Estienne M Reho P Matricardi S Castellotti B Visser WE Zuffardi O & Visser TJ. Clinical and molecular characteristics of SLC16A2 (MCT8) mutations in three families with the Allan-Herndon-Dudley syndrome. Human Mutation 2017 38 260–264. (10.1002/humu.23140) 27805744

[110] Anik A Kersseboom S Demir K Catli G Yis U Bober E van Mullem A van Herebeek REA Hız S Abacı A, *et al.* Psychomotor retardation caused by a defective thyroid hormone transporter: report of two families with different MCT8 mutations. Hormone Research in Paediatrics 2014 82 261–271. (10.1159/000365191) 25247785

[111] Papadimitriou A Dumitrescu AM Papavasiliou A Fretzayas A Nicolaidou P & Refetoff S. A novel monocarboxylate transporter 8 gene mutation as a cause of severe neonatal hypotonia and developmental delay. Pediatrics 2008 121 e199–e202. (10.1542/peds.2007-1247) 18166539

[112] Kakinuma H Itoh M & Takahashi H. A novel mutation in the monocarboxylate transporter 8 gene in a boy with putamen lesions and low free T4 levels in cerebrospinal fluid. Journal of Pediatrics 2005 147 552–554. (10.1016/j.jpeds.2005.05.012) 16227048

[113] Herzovich V Vaiani E Marino R Dratler G Lazzati JM Tilitzky S Ramirez P Lorcansky S Rivarola MA & Belgorosky A. Unexpected peripheral markers of thyroid function in a patient with a novel mutation of the MCT8 thyroid hormone transporter gene. Hormone Research 2007 67 1–6. (10.1159/000095805) 16974106

[114] Crushell E & Reardon W. Elevated TSH levels in a mentally retarded boy. European Journal of Pediatrics 2010 169 573–575. (10.1007/s00431-009-1075-0) 19936787

[115] Namba N Etani Y Kitaoka T Nakamoto Y Nakacho M Bessho K Miyoshi Y Mushiake S Mohri I Arai H, *et al.* Clinical phenotype and endocrinological investigations in a patient with a mutation in the MCT8 thyroid hormone transporter. European Journal of Pediatrics 2008 167 785–791. (10.1007/s00431-007-0589-6) 17899191

[116] Garcia-de Teresa B Gonzalez-Del Angel A Reyna-Fabian ME Ruiz-Reyes Mde L Calzada-Leon R Perez-Enriquez B & Alcántara-Ortigoza MA. Deletion of exon 1 of the SLC16A2 gene: a common occurrence in patients with Allan-Herndon-Dudley syndrome. Thyroid 2015 25 361–367. (10.1089/thy.2014.0284) 25517855

[117] Zung A Visser TJ Uitterlinden AG Rivadeneira F & Friesema ECH. A child with a deletion in the monocarboxylate transporter 8 gene: 7-year follow-up and effects of thyroid hormone treatment. European Journal of Endocrinology 2011 165 823–830. (10.1530/EJE-11-0358) 21896621

[118] Filho HCM Marui S Manna TD Brust ES Radonsky V Kuperman H Dichtchekenian V Setian N & Damiani D. Novel mutation in MCT8 gene in a Brazilian boy with thyroid hormone resistance and severe neurologic abnormalities. Arquivos Brasileiros de Endocrinologia e Metabologia 2011 55 60–66. (10.1590/s0004-27302011000100008) 21468521

[119] Wemeau JL Pigeyre M Proust-Lemoine E d'Herbomez M Gottrand F Jansen J Visser TJ & Ladsous M. Beneficial effects of propylthiouracil plus L-thyroxine treatment in a patient with a mutation in MCT8. Journal of Clinical Endocrinology and Metabolism 2008 93 2084–2088. (10.1210/jc.2007-2719) 18334584

[120] Visser WE Vrijmoeth P Visser FE Arts WFM van Toor H & Visser TJ. Identification, functional analysis, prevalence and treatment of monocarboxylate transporter 8 (MCT8) mutations in a cohort of adult patients with mental retardation. Clinical Endocrinology 2013 78 310–315. (10.1111/cen.12023) 22924588

[121] Gika AD Siddiqui A Hulse AJ Edward S Fallon P McEntagart ME Jan W Josifova D Lerman-Sagie T Drummond J, *et al.* White matter abnormalities and dystonic motor disorder associated with mutations in the SLC16A2 gene. Developmental Medicine and Child Neurology 2010 52 475–482. (10.1111/j.1469-8749.2009.03471.x) 19811520 PMC5800746

[122] Verge CF Konrad D Cohen M Di Cosmo C Dumitrescu AM Marcinkowski T Hameed S Hamilton J Weiss RE & Refetoff S. Diiodothyropropionic acid (DITPA) in the treatment of MCT8 deficiency. Journal of Clinical Endocrinology and Metabolism 2012 97 4515–4523. (10.1210/jc.2012-2556) 22993035 PMC3513545

[123] van Geest FS Groeneweg S van den Akker ELT Bacos I Barca D van den Berg SAA Bertini E Brunner D Brunetti-Pierri N Cappa M, *et al.* Long-term efficacy of T3 analogue Triac in children and adults with MCT8 deficiency: a real-life retrospective cohort study. Journal of Clinical Endocrinology and Metabolism 2022 107 e1136–e1147. (10.1210/clinem/dgab750) 34679181 PMC8852204

[124] Groeneweg S Peeters RP Moran C Stoupa A Auriol F Tonduti D Dica A Paone L Rozenkova K Malikova J, *et al.* Effectiveness and safety of the tri-iodothyronine analogue Triac in children and adults with MCT8 deficiency: an international, single-arm, open-label, phase 2 trial. Lancet. Diabetes and Endocrinology 2019 7 695–706. (10.1016/S2213-8587(1930155-X) 31377265 PMC7611958

[125] Refetoff S Pappa T Williams MK Matheus MG Liao XH Hansen K Nicol L Pierce M Blasco PA Wiebers Jensen M, *et al.* Prenatal treatment of thyroid hormone cell membrane transport defect caused by MCT8 gene mutation. Thyroid 2021 31 713–720. (10.1089/thy.2020.0306) 32746752 PMC8110025

[126] Dumitrescu AM Liao XH Abdullah MSY La do-Abeal J Majed FA Moeller LC Boran G Schomburg L Weiss RE & Refetoff S. Mutations in SECISBP2 result in abnormal thyroid hormone metabolism. Nature Genetics 2005 37 1247–1252. (10.1038/ng1654) 16228000

[127] Schoenmakers E Carlson B Agostini M Moran C Rajanayagam O Bochukova E Tobe R Peat R Gevers E Muntoni F, *et al.* Mutation in human selenocysteine transfer RNA selectively disrupts selenoprotein synthesis. Journal of Clinical Investigation 2016 126 992–996. (10.1172/JCI84747) 26854926 PMC4767355

[128] Geslot A Savagner F & Caron P. Inherited selenocysteine transfer RNA mutation: clinical and hormonal evaluation of 2 patients. European Thyroid Journal 2021 10 542–547. (10.1159/000518275) 34956927 PMC8647050

[129] Schoenmakers E & Chatterjee K. Human genetic disorders resulting in systemic selenoprotein deficiency. International Journal of Molecular Sciences 2021 22 12927. (10.3390/ijms222312927) 34884733 PMC8658020

[130] Di Cosmo C McLellan N Liao XH Khanna KK Weiss RE Papp L & Refetoff S. Clinical and molecular characterization of a novel selenocysteine insertion sequence-binding protein 2 (SBP2) gene mutation (R128X). Journal of Clinical Endocrinology and Metabolism 2009 94 4003–4009. (10.1210/jc.2009-0686) 19602558 PMC2758735

[131] Hamajima T Mushimoto Y Kobayashi H Saito Y & Onigata K. Novel compound heterozygous mutations in the SBP2 gene: characteristic clinical manifestations and the implications of GH and triiodothyronine in longitudinal bone growth and maturation. European Journal of Endocrinology 2012 166 757–764. (10.1530/EJE-11-0812) 22247018

[132] Fu J Korwutthikulrangsri M Gönç EN Sillers L Liao XH Alikaşifoğlu A Kandemir N Menucci MB Burman KD Weiss RE, *et al.* Clinical and molecular analysis in 2 families with novel compound heterozygous SBP2 (SECISBP2) mutations. Journal of Clinical Endocrinology and Metabolism 2020 105 e6–e11. (10.1210/clinem/dgz169) 32084277 PMC7034949

[133] Schoenmakers E Marelli F Jørgensen HF Visser WE Moran C Groeneweg S Avalos C Jurgens SJ Figg N Finigan A, *et al.* Selenoprotein deficiency disorder predisposes to aortic aneurysm formation. Nature Communications 2023 14 7994. (10.1038/s41467-023-43851-6) PMC10693596 38042913

[134] Azevedo MF Barra GB Naves LA Ribeiro Velasco LF Godoy Garcia Castro P de Castro LCG Amato AA Miniard A Driscoll D Schomburg L, *et al.* Selenoprotein-related disease in a young girl caused by nonsense mutations in the SBP2 gene. Journal of Clinical Endocrinology and Metabolism 2010 95 4066–4071. (10.1210/jc.2009-2611) 20501692

[135] Schoenmakers E Agostini M Mitchell C Schoenmakers N Papp L Rajanayagam O Padidela R Ceron-Gutierrez L Doffinger R Prevosto C, *et al.* Mutations in the selenocysteine insertion sequence-binding protein 2 gene lead to a multisystem selenoprotein deficiency disorder in humans. Journal of Clinical Investigation 2010 120 4220–4235. (10.1172/JCI43653) 21084748 PMC2993594

[136] Çatli G Fujisawa H Kirbiyik Ö Mimoto MS Gençpinar P Özdemir TR Dündar BN & Dumitrescu AM. A novel homozygous selenocysteine insertion sequence binding Protein 2 (SECISBP2, SBP2) gene mutation in a Turkish boy. Thyroid 2018 28 1221–1223. (10.1089/thy.2018.0015) 29882503 PMC6154453

[137] Schomburg L Dumitrescu AM Liao XH Bin-Abbas B Hoeflich J Köhrle J & Refetoff S. Selenium supplementation fails to correct the selenoprotein synthesis defect in subjects with SBP2 gene mutations. Thyroid 2009 19 277–281. (10.1089/thy.2008.0397) 19265499 PMC2858371

[138] Saito Y Shichiri M Hamajima T Ishida N Mita Y Nakao S Hagihara Y Yoshida Y Takahashi K Niki E, *et al.* Enhancement of lipid peroxidation and its amelioration by vitamin E in a subject with mutations in the SBP2 gene. Journal of Lipid Research 2015 56 2172–2182. (10.1194/jlr.M059105) 26411970 PMC4617404

[139] Schneider MJ Fiering SN Thai B Wu SY St Germain E Parlow AF St Germain DL & Galton VA. Targeted disruption of the type 1 selenodeiodinase gene (Dio1) results in marked changes in thyroid hormone economy in mice. Endocrinology 2006 147 580–589. (10.1210/en.2005-0739) 16223863

[140] Schneider MJ Fiering SN Pallud SE Parlow AF St Germain DL & Galton VA. Targeted disruption of the type 2 selenodeiodinase gene (DIO2) results in a phenotype of pituitary resistance to T4. Molecular Endocrinology 2001 15 2137–2148. (10.1210/mend.15.12.0740) 11731615

[141] Hernandez A Martinez ME Fiering S Galton VA & St Germain D. Type 3 deiodinase is critical for the maturation and function of the thyroid axis. Journal of Clinical Investigation 2006 116 476–484. (10.1172/JCI26240) 16410833 PMC1326144

[142] Liao XH Di Cosmo C Dumitrescu AM Hernandez A Van Sande J St Germain DL Weiss RE Galton VA & Refetoff S. Distinct roles of deiodinases on the phenotype of Mct8 defect: a comparison of eight different mouse genotypes. Endocrinology 2011 152 1180–1191. (10.1210/en.2010-0900) 21285310 PMC3040057

[143] Franca MM German A Fernandes GW Liao XH Bianco AC Refetoff S & Dumitrescu AM. Human type 1 iodothyronine deiodinase (DIO1) mutations cause abnormal thyroid hormone metabolism. Thyroid 2021 31 202–207. (10.1089/thy.2020.0253) 32718224 PMC7891200

[144] Furman AE Hannoush Z Echegoyen FB Dumitrescu A Refetoff S & Weiss RE. Novel DI01 gene mutation acting as phenotype modifier for novel compound heterozygous TPO gene mutations causing congenital hypothyroidism. Thyroid 2021 31 1589–1591. (10.1089/thy.2021.0210) 34128397 PMC8917882

