# Supplementary material for: 2024 European Thyroid Association Guidelines on diagnosis and management of genetic disorders of thyroid hormone transport, metabolism and action
Source: Eur Thyroid J. 2024 Aug 3;13(4):e240125. doi: 10.1530/ETJ-24-0125 (PMC11301568; doi:10.1530/ETJ-24-0125)
Supplement: Supplementary Figure 1: Compilation of known pathogenic variants in THRB. Domains of TRβ2 and TRβ1 showing that with two exceptions (light blue circles) all pathogenic TRβ variants described to date, localise to three clusters within the hormone binding domain. [file supplementary_figure_1.pdf]

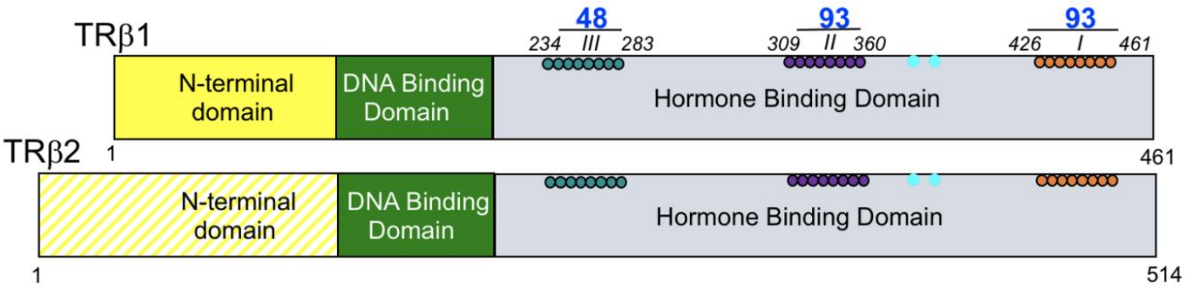

Cluster III 234-283		Cluster II 306-360		Cluster I 426-461		Outside Clusters
A234D/T/V	A268D/G	K306Q	A335P	T426I	C446G/R/S/*	Q374K
Q235R	F269V	G307D	V336M/L	L428V	P447T	R383C/H/S
W239R	H271D	C309Y	T337A/Δ	R429Q/W	T448Hfs*17	
Q241P	F272V/C	M310I/L/T/V	R338L/P/Q/W	M430Δ	E449X/Dfs*12	
R243P/Q/W	T273R	E311K	Q340E/H	I431L/M/T/V/fs	L450H/P/V	
L246P/V	I276L/N/Δ	M313T/V	L341P/V	G432X/Δ	F451C/I/L/S/V/*/fs463	
P247L/S	T277A/I	S314C/F/Y	K342I	A433Cfs*29	P452Tfs*15, P452Sfs*13	
I250F/T	P278L	R316C/H	N343K	C434*	P452H/I/L/R/S/Δ	
G251E/R/V	A279E/V	A317D/S/T/V	G344A/E	H435L/N/P/Q/R/Y	P453A/H/L/N/R/S/T/Y	
G261Δ	I280S/V	R320C/G/H/L/P/S	G345C/D/R/S/V	A436Vfs*29	L454S/V/W/ */Ffs*11	
V264A/D/F/L	R282G/K/S/T	Y321C/S	L346F/R/V	R438C/H/P/Afs*5	F455I/L/S	
D265A/G/Y	V283A	D322A/G/H/N	G347A/E/R/W	F439L	L456S	
		T327A/I/P/S	V348E	L440P	E457A/D/G/K/Q/Rfs*8	
		L328S	V349A/E/M	H441Tfs*2	V458A/E/G	
		T329I/N/S	S350L/P	M442T/V	F459C/L/V	
		L330S	D351G	K443E/N	E460K/G	
		N331D/H/K	I353F/M/T/V	E445G/K/*	D461G	
		G332E/R	F354L			
		E333D/K/Q	G357R			
		M334R/T	L360M			

Supplementary Figure 1: Compilation of known pathogenic variants in *THRB*.
Domains of TRβ2 and TRβ1 showing that with two exceptions (light blue circles) all pathogenic TRβ variants described to date, localise to three clusters within the hormone binding domain.
