# Supplementary material for: 2024 European Thyroid Association Guidelines on diagnosis and management of genetic disorders of thyroid hormone transport, metabolism and action
Source: Eur Thyroid J. 2024 Aug 3;13(4):e240125. doi: 10.1530/ETJ-24-0125 (PMC11301568; doi:10.1530/ETJ-24-0125)
Supplement: Supplementary Figure 2: Compilation of known pathogenic variants in MCT8 (SLC16A2). Pathogenic variants in MCT8 (SLC16A2). Different coloured boxes depict the location of variants in transmembrane domains (TMDs) or extracellular (top) and intracellular (bottom) loops of the protein. Large deletions  [file supplementary_figure_2.pdf]

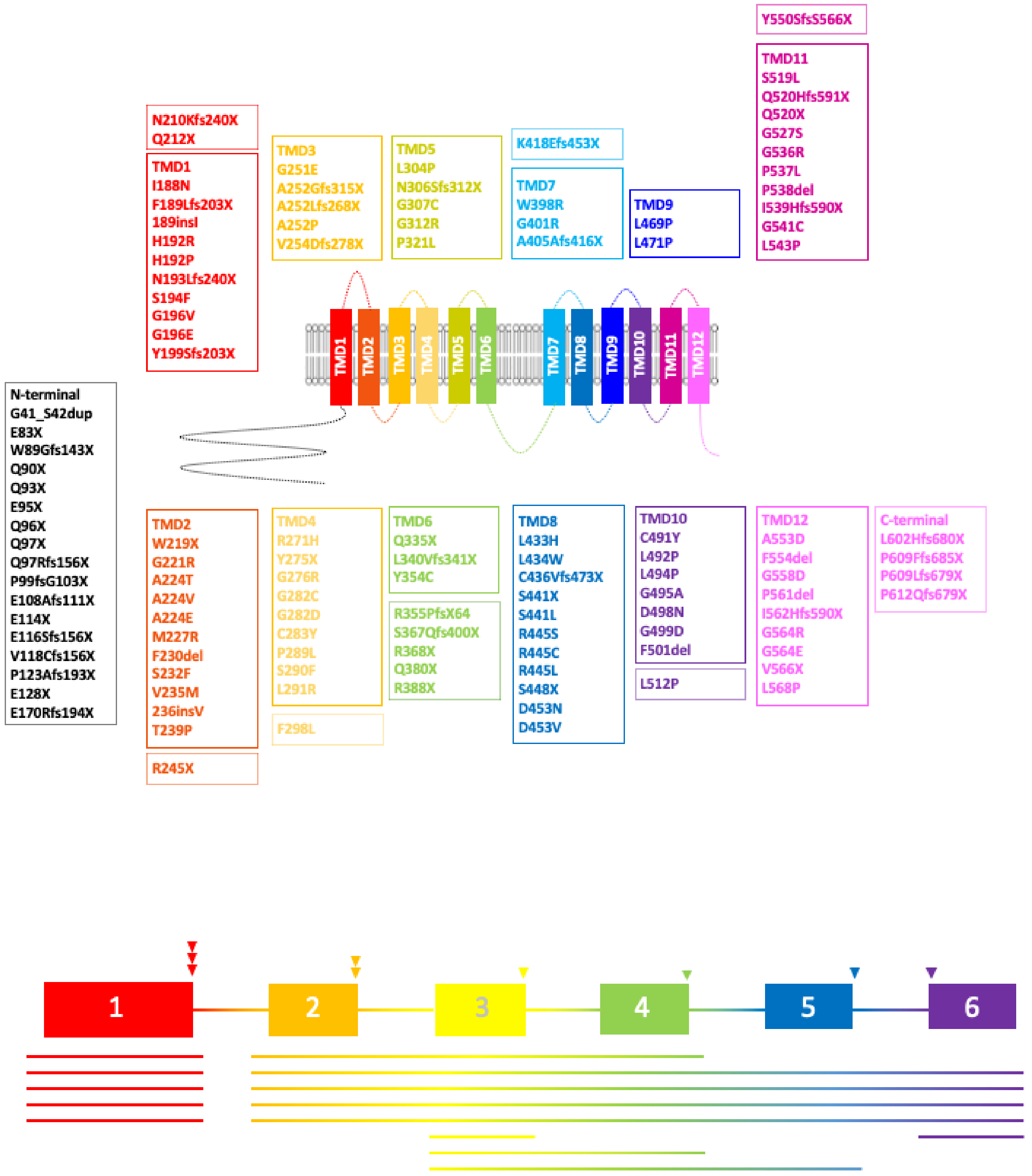

Supplementary Figure 2: Compilation of known pathogenic variants in MCT8 (*SLC16A2*). Pathogenic variants in MCT8 (*SLC16A2*). Different coloured boxes depict the location of variants in transmembrane domains (TMDs) or extracellular (top) and intracellular (bottom) loops of the protein. Large deletions (lines) and splice site variants (arrowheads) are superimposed on the genomic organisation or *SLC16A2* (bottom of picture).
