# Supplementary material for: 2024 European Thyroid Association Guidelines on diagnosis and management of genetic disorders of thyroid hormone transport, metabolism and action
Source: Eur Thyroid J. 2024 Aug 3;13(4):e240125. doi: 10.1530/ETJ-24-0125 (PMC11301568; doi:10.1530/ETJ-24-0125)
Supplement: Supplementary Figure 3: Phenotype of known DIO1 mutations. Serum rT3 concentrations and rT3/T3 ratios in affected (closed circles) and unaffected (open circles) members of two families with DIO1 mutations. Deiodinase enzyme activity, measured in cells expressing either the WT and two D1 mutants alon [file supplementary_figure_3.pdf]

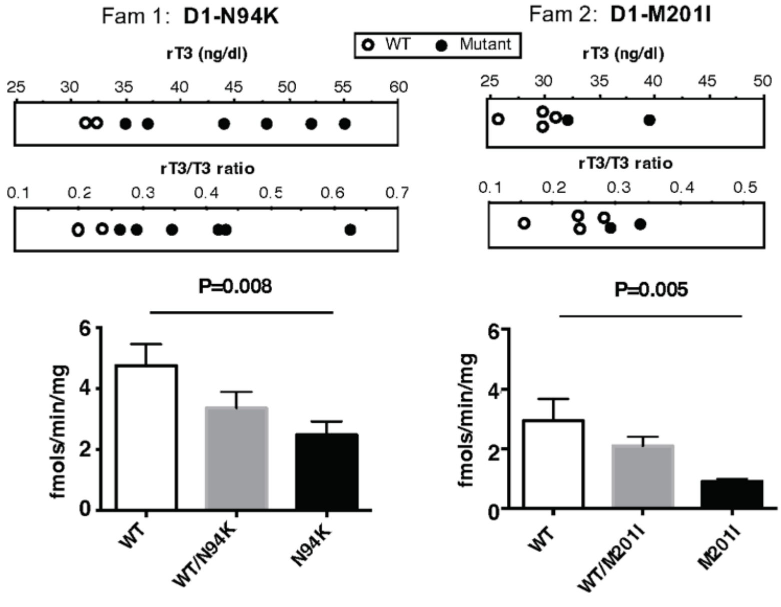

Supplementary Figure 3: Phenotype of known *DIO1* mutations.

Serum rT3 concentrations and rT3/T3 ratios in affected (closed circles) and unaffected (open circles) members of two families with DIO1 mutations. Deiodinase enzyme activity, measured in cells expressing either the WT and two D1 mutants alone or in combination with WT (simulating the heterozygous status of patients), is shown.
