# Supplementary material for: 2024 European Thyroid Association Guidelines on diagnosis and management of genetic disorders of thyroid hormone transport, metabolism and action
Source: Eur Thyroid J. 2024 Aug 3;13(4):e240125. doi: 10.1530/ETJ-24-0125 (PMC11301568; doi:10.1530/ETJ-24-0125)
Supplement: Supplementary Table 1: Compilation of all published pathogenic variants in THRA [file supplementary_table_1.pdf]

Supplementary Table 1: Compilation of all published pathogenic variants in *THRA*

THRA Mutation	Number of cases (families)	TR α protein(s) affected	Reference(s)
G207E	2 (1)	TR α1 and α2	(1)
D211G	2 (1)	TR α1 and α2	(2)
M256T	1 (1)	TR α1 and α2	(3)
M259T	1 (1)	TR α1 and α2	(4)
A263S	7 (1)	TR α1 and α2	(5)
A263V	8 (3)	TR α1 and α2	(6), (7)
T273A	1 (1)	TR α1 and α2	(4)
L274P	1 (1)	TR α1 and α2	(8)
G291S	2 (2)	TR α1 and α2	(9), (10)
N359Y	1 (1)	TR α1 and α2	(11)
C380SfsX9	1 (1)	TR α1 only	(12)
C380fs387X	1 (1)	TR α1 only	(5)
A382PfsX7	1 (1)	TR α1 only	(13)
R384C	1 (1)	TR α1 only	(14)
R384H	2 (1)	TR α1 only	(5)
C392X	1 (1)	TR α1 only	(15)
E395X	1 (1)	TR α1 only	(16)
F397fs406X	2 (1)	TR α1 only	(17)
P398R	1 (1)	TR α1 only	(15)
E403K	2 (1)	TR α1 only	(15)
E403X	2 (2)	TR α1 only	(15), (18)

References

1. Van Gucht ALM, Moran C, Meima ME, Visser WE, Chatterjee K, Visser TJ & Peeters RP. Resistance to thyroid hormone due to heterozygous mutation in thyroid hormone receptor alpha. *Current Topics in Developmental Biology* 2017 **125** 337–355. (https://doi.org/10.1016/bs.ctdb.2017.02.001)

2. Van Gucht ALM, Meima ME, Zwaveling-Soonawala N, Visser WE, Fliers E, Wennink JMB, Henny C, Visser TJ, Peeters RP & van Trotsenburg ASP. Resistance to thyroid hormone alpha in an 18-month-old girl: clinical, therapeutic and molecular characteristics. *Thyroid* 2016 **26** 338–346. (https://doi.org/10.1089/thy.2015.0463)

3. Wejaphikul K, Groeneweg S, Hilhorst-Hofsee Y, Chatterjee VK, Peeters RP, Meima ME & Visser WE. Insight into molecular determinants of T3 versus T4 recognition from mutations in thyroid hormone receptor alpha and beta. *Journal of Clinical Endocrinology and Metabolism* 2019 **104** 3491-3500. (https://doi.org/10.1210/jc.2018-02794)

4. Le Maire A, Bouhours-Nouet N, Soamalala J, Mirebeau-Prunier D, Paloni M, Guee L, Heron D, Mignot C, Illouz F, Joubert F, *et al.* Two novel cases of resistance to thyroid hormone due to THRA mutation. *Thyroid* 2020 **30** 1217–1221. (https://doi.org/10.1089/ thy.2019.0602)

5. Demir K, van Gucht ALM, Buyukinan M, Catli G, Ahan Y, Bas VN, Dundar B, Ozkan B, Meima ME, Visser WE, *et al.* Diverse genotypes and phenotypes of three novel thyroid hormone receptor alpha mutations. *Journal of Clinical Endocrinology and Metabolism* 2016 **101** 2945–2954. (https://doi.org/10.1210/jc.2016-1404)

6. Moran C, Agostini M, Visser E, Schoenmakers E, Schoenmakers N, Offiah AC, Poole K, Rajanayagam O, Lyons G, Halsall D, *et al.* Resistance to thyroid hormone caused by a mutation in thyroid hormone receptor (TR) alpha1 and alpha2: clinical, biochemical and genetic analyses of three related patients. *Lancet Diabetes and Endocrinology* 2014 **2** 619–626. (https://doi.org/10.1016/S2213-8587(14)70111-1)

7. Dahll LK, Westbye AB, Vinorum K, Sejersted Y, Barøy T, Thorsby PM & Hammerstad SS. Clinical and Biochemical characteristics of untreated adult patients with resistance to thyroid hormone alpha. *Journal of the Endocrine Society* 2023 **7** bvad089. (https://doi.org/10.1210/jendso/bvad089)

8. Moran C, Agostini M, McGowan A, Schoenmakers E, Fairall L, Lyons G, Rajanayagam O, Watson L, Offiah A, Barton J, *et al.* Contrasting phenotype in resistance to thyroid hormone alpha correlate with divergent properties of thyroid hormone receptor a1 mutant proteins. *Thyroid* 2017 **27** 973–982. (https://doi.org/10.1089/thy.2017.0157)

9. Korkmaz O, Ozen S, Ozdemir TR, Goksen D & Darcan S. A novel thyroid hormone receptor alpha gene mutation, clinic characteristics, and follow-up findings in a patient with thyroid hormone resistance. *Hormones* 2019 **18** 223–227. (https://doi.org/10.1007/s42000-019-00094-9)

10. Al Shidhani A, Ullah I, AlSaffar H, Kindi AA, Al Nabhani H & Al Yaarubi S. Thyroid hormone resistance due to a novel de novo mutation in thyroid hormone receptor alpha: first case report from the Middle East and North Africa. *Oman Medical Journal* 2021 **36** e226. (https://doi.org/10.5001/omj.2021.20)

11. Espiard S, Savagner F, Flamant F, Vlaeminck-Guillem V, Guyot R, Munier M, d'Herbomez M, Bourguet W, Pinto G, Rose C, *et al.* A novel mutation in THRA gene associated with an atypical phenotype of resistance to thyroid hormone. *Journal of Clinical Endocrinology and Metabolism* 2015 **100** 2841–2848. (https://doi.org/10.1210/jc.2015-1120)

12. Furman AE, Dumitrescu AM, Refetoff S & Weiss RE. Early diagnosis and treatment of an infant with a novel thyroid hormone receptor alpha gene (cC380SfsX9) mutation. *Thyroid* 2021 **31** 1003–1005. (https://doi.org/10.1089/thy.2020.0695)

13. Moran C, Schoenmakers N, Agostini M, Schoenmakers E, Offiah A, Kydd A, Kahaly G, Mohr-Kahaly S, Rajanayagam O, Lyons G, *et al.* An adult female with resistance to thyroid hormone mediated by

defective thyroid hormone receptor α. *Journal of Clinical Endocrinology and Metabolism* 2013 **98** 4254–4261. (https://doi.org/10.1210/jc.2013-22155)

14. Yuen RKC, Thiruvahindrapuram B, Merico D, Walker S, Tammimies K, Hoang N, Chrysler C, Nalpathamkalam T, Pellecchia G, Liu Y, *et al.* Whole genome sequencing of Quartet families with autism spectrum disorder. *Nature Medicine* 2015 **21** 185–191. (https://doi.org/10.1038/nm.3792)

15. Tylki-Szymańska A, Acuna-Hidalgo R, Krajewska-Walasek M, Lecka- Ambroziak A, Steehouwer M, Gilissen C, Brunner HG, Jurecka A, Różdżyńska-Świątkowska A, Hoischen A , *et al.* Thyroid hormone resistance syndrome due to mutations in the thyroid hormone receptor α gene (THRA). *Journal of Medical Genetics* 2015 **52** 312–316. (https://doi.org/10.1136/jmedgenet-2014-102936)

16. Sun H, Wu H, Xie R, Wang F, Chen T, Chen X, Wang X, Flamant F & Chen L. New case of thyroid hormone resistance a caused by a mutation of THRA/Tra1. *Journal of the Endocrine Society* 2019 **3** 665–669. (https://doi.org/10.1210/js.2019-00011)

17. van Mullem A, van Heerebeek R, Chrysis D, Visser E, Medici M, Andrikoula M, Tsatsoulis A, Peeters R & Visser TJ. Clinical phenotype and mutant TRα1. *New England Journal of Medicine* 2012 **366** 1451–1453. (https://doi.org/10.1056/NEJMc1113940)

18. Bochukova E, Schoenmakers N, Agostini M, Schoenmakers E, Rajanayagam O, Keogh JM, Henning E, Reinemund J, Gevers E, Sarri M, *et al.* A mutation in the thyroid hormone receptor alpha gene. *New England Journal of Medicine* 2012 **366** 243–249. (https://doi.org/10.1056/NEJMoa1110296)
