# Supplementary material for: 2024 European Thyroid Association Guidelines on diagnosis and management of genetic disorders of thyroid hormone transport, metabolism and action
Source: Eur Thyroid J. 2024 Aug 3;13(4):e240125. doi: 10.1530/ETJ-24-0125 (PMC11301568; doi:10.1530/ETJ-24-0125)
Supplement: Supplementary Table 2: Compilation of all published pathogenic variants in SECISBP2 and TRU-TCA1-1 [file supplementary_table_2.pdf]

**Supplementary Table 2**: Compilation of all published pathogenic variants in *SECISBP2* and *TRU-TCA1-1*

| | | Number of cases (families) | Aminoacid changes | Reference |
|---|---|---|---|---|
| **SECISBP2 Mutation(s)** | | | | |
| | Biallelic c.1619 G>A | 3(1) | *R540Q* | (1) |
| | c.1312A>T<br>IVS8ds+29 G>A | 1(1) | K438X missplicing and fs431X | (1) |
| | Biallelic c.382 C > T | 1(1) | *R128X* | (2), (3) |
| | c.358 C>T<br>c.2308 C>T | 1(1) | R120X<br>R770X | (4) |
| | c. 2017T > C<br>1–5 intronic SNP's | 1(1) | C691R<br>fs65X + fs76X | (5) |
| | c.668delT<br>IVS7 -155, T>A | 1(1) | F223fs255X<br>fs295X + fs302X | (5), (3) |
| | c.1529_1541dup CCAGCGCCCCACT<br>c.235 C>T | 1(1) | M515fs563X<br>Q79X | (6) |
| | Biallelic c.800_801insA | 1(1) | *K267Kfs*2* | (7) |
| | c.2344 C>T<br>c.2045-2048 delAACA | 1(1) | Q782X<br>K682fs683X | (8) |
| | c.589C>T<br>c.2037G > T | 1(1) | R197X<br>E679D | (8) |
| | c.1312A>T<br>c.1894-3C>G | 1(1) | K438X<br>splicing exon 13-14<br>fs631X | (3) |
| | Biallelic Chr 9: 91935388-91941101del | 1(1) | *E61E*4* | (3) |
| | c.1588A>G<br>c1711C>T | 2(1) | T530A<br>Q571X | (9) |
| | c.283del<br>c.589C>T | 1(1) | Y95Ifs*31<br>R197X | (9) |
| | c.838_839del<br>c.2091C>A | 1(1) | V280Nfs*3<br>N697K | (9) |
| | Biallelic c.1089+1 G>C | 1(1) | Unknown | (9) |
| | Biallelic c.1089+1 G>T | 1(1) | Unknown | (9) |
| | Biallelic c.358C>T | 1(1) | *R120X* | (9) |
| ***TRU-TCA 1-1* mutation** | | | | |
| | Biallelic c.65 G>C | 1(1) | | (10) |
| | Biallelic c.65 G>C | 1(1) | | (11) |

## References


1. Dumitrescu AM, Liao XH, Abdullah MSY, La do-Abeal J, Majed FA, Moeller LC, Boran G, Schomburg L, Weiss RE & Refetoff S. Mutations in SECISBP2 result in abnormal thyroid hormone metabolism. *Nature Genetics* 2005 **37** 1247–1252. (https://doi.org/10.1038/ng1654)

2. Di Cosmo C, McLellan N, Liao XH, Khanna KK, Weiss RE, Papp L & Refetoff S. Clinical and molecular characterization of a novel selenocysteine insertion sequence-binding protein 2 (SBP2) gene mutation (R128X). *Journal of Clinical Endocrinology and Metabolism* 2009 **94** 4003–4009. (https://doi.org/10.1210/jc.2009-0686)

3. Schoenmakers E, Marelli F, Jørgensen HF, Visser WE, Moran C, Groeneweg S, Avalos C, Jurgens SJ, Figg N, Finigan A, et al. Selenoprotein deficiency disorder predisposes to aortic aneurysm formation. *Nature Communications* 2023 **14** 7994. (https://doi.org/10.1038/s41467-023-43851-6)

4. Azevedo MF, Barra GB, Naves LA, Ribeiro Velasco LF, Godoy Garcia Castro P, de Castro LCG, Amato AA, Miniard A, Driscoll D, Schomburg L, et al. Selenoprotein-related disease in a young girl caused by nonsense mutations in the SBP2 gene. *Journal of Clinical Endocrinology and Metabolism* 2010 **95** 4066–4071. (https://doi.org/10.1210/jc.2009-2611)

5. Schoenmakers E, Agostini M, Mitchell C, Schoenmakers N, Papp L, Rajanayagam O, Padidela R, Ceron-Gutierrez L, Doffinger R, Prevosto C, et al. Mutations in the selenocysteine insertion sequence-binding protein 2 gene lead to a multisystem selenoprotein deficiency disorder in humans. *Journal of Clinical Investigation* 2010 **120** 4220–4235. (https://doi.org/10.1172/JCI43653)

6. Hamajima T, Mushimoto Y, Kobayashi H, Saito Y & Onigata K. Novel compound heterozygous mutations in the SBP2 gene: characteristic clinical manifestations and the implications of GH and triiodothyronine in longitudinal bone growth and maturation. *European Journal of Endocrinology* 2012 **166** 757–764. (https://doi.org/10.1530/EJE-11-0812)

7. Çatli G, Fujisawa H, Kirbiyik Ö, Mimoto MS, Gençpinar P, Özdemir TR, Dündar BN & Dumitrescu AM. A novel homozygous selenocysteine insertion sequence binding Protein 2 (SECISBP2, SBP2) gene mutation in a Turkish boy. *Thyroid* 2018 **28** 1221–1223. (https://doi.org/10.1089/thy.2018.0015)

8. Fu J, Korwutthikulrangsri M, Gönç EN, Sillers L, Liao XH, Alikaşifoğlu A, Kandemir N, Menucci MB, Burman KD, Weiss RE, et al. Clinical and molecular analysis in 2 families with novel compound heterozygous SBP2 (SECISBP2) mutations. *Journal of Clinical Endocrinology and Metabolism* 2020 **105** e6–e11. (https://doi.org/10.1210/clinem/dgz169)

9. Stoupa A, Franca MM, Abdulhadi-Atwan M, Fuijisawa H, Korwutthikulrangsri M, Marchand I, Polak G, Beltrand J, Polak M, Kariyawasam D, et al. Severe neurodevelopmental phenotype, diagnostic and treatment challenges in patients with SECISBP2 (SBP2) deficiency. *Genetics in Medicine* 2024 In Press.

10. Schoenmakers E, Carlson B, Agostini M, Moran C, Rajanayagam O, Bochukova E, Tobe R, Peat R, Gevers E, Muntoni F, et al. Mutation in human selenocysteine transfer RNA selectively disrupts selenoprotein synthesis. *Journal of Clinical Investigation* 2016 **126** 992–996. (https://doi.org/10.1172/JCI84747)

11. Geslot A, Savagner F & Caron P. Inherited selenocysteine transfer RNA mutation: clinical and hormonal evaluation of 2 patients. *European Thyroid Journal* 2021 **10** 542–547. (https://doi.org/10.1159/000518275)
