# Supplementary material for: 2024 European Thyroid Association Guidelines on diagnosis and management of genetic disorders of thyroid hormone transport, metabolism and action
Source: Eur Thyroid J. 2024 Aug 3;13(4):e240125. doi: 10.1530/ETJ-24-0125 (PMC11301568; doi:10.1530/ETJ-24-0125)
Supplement: Supplementary Table 3: Phenotype of mice deficient in deiodinases [file supplementary_table_3.pdf]

Supplementary Table 3: Phenotype of mice deficient in deiodinases

KO models	Dio1-KO	Dio2-KO	Dio3-KO	Secisbp2-KO
**Serum T4**	Elevated	Normal /? Elevated	Decreased	Elevated
**Serum T3**	Normal	Normal	Decreased	Normal
**Serum rT3**	Elevated	Normal /? Elevated	NR	Elevated
**TSH**	Normal	Elevated	Normal or slightly elevated	Elevated
**Systemic phenotype**	Euthyroid	Euthyroid	Hypothyroid	Hypothyroidism
**Reproduction**	Normal	Normal	Impaired	Impaired
